# A Bioinspired *in vitro* Lung Model to Study Particokinetics of Nano-/Microparticles Under Cyclic Stretch and Air-Liquid Interface Conditions

**DOI:** 10.3389/fbioe.2021.616830

**Published:** 2021-01-29

**Authors:** Ali Doryab, Mehmet Berat Taskin, Philipp Stahlhut, Andreas Schröppel, Sezer Orak, Carola Voss, Arti Ahluwalia, Markus Rehberg, Anne Hilgendorff, Tobias Stöger, Jürgen Groll, Otmar Schmid

**Affiliations:** ^1^Comprehensive Pneumology Center Munich, Member of the German Center for Lung Research, Munich, Germany; ^2^Helmholtz Zentrum München—German Research Center for Environmental Health, Institute of Lung Biology and Disease, Munich, Germany; ^3^Department of Functional Materials in Medicine and Dentistry, Bavarian Polymer Institute, University of Würzburg, Würzburg, Germany; ^4^Research Center “E. Piaggio”, University of Pisa, Pisa, Italy; ^5^Department of Information Engineering, University of Pisa, Pisa, Italy; ^6^Center for Comprehensive Developmental Care (CDeC^LMU^), Dr. von Haunersches Children's Hospital University, Hospital of the Ludwig-Maximilians University, Munich, Germany

**Keywords:** lung cell model, cyclic stretch, ALI culture, bioinspired membrane, particle study

## Abstract

Evolution has endowed the lung with exceptional design providing a large surface area for gas exchange area (ca. 100 m^2^) in a relatively small tissue volume (ca. 6 L). This is possible due to a complex tissue architecture that has resulted in one of the most challenging organs to be recreated in the lab. The need for realistic and robust *in vitro* lung models becomes even more evident as causal therapies, especially for chronic respiratory diseases, are lacking. Here, we describe the **C**yclic ***I****n* ***VI****tro* **C**ell-stretch (CIVIC) “breathing” lung bioreactor for pulmonary epithelial cells at the air-liquid interface (ALI) experiencing cyclic stretch while monitoring stretch-related parameters (amplitude, frequency, and membrane elastic modulus) under real-time conditions. The previously described biomimetic copolymeric BETA membrane (5 μm thick, bioactive, porous, and elastic) was attempted to be improved for even more biomimetic permeability, elasticity (elastic modulus and stretchability), and bioactivity by changing its chemical composition. This biphasic membrane supports both the initial formation of a tight monolayer of pulmonary epithelial cells (A549 and 16HBE14o^−^) under submerged conditions and the subsequent cell-stretch experiments at the ALI without preconditioning of the membrane. The newly manufactured versions of the BETA membrane did not improve the characteristics of the previously determined optimum BETA membrane (9.35% PCL and 6.34% gelatin [w/v solvent]). Hence, the optimum BETA membrane was used to investigate quantitatively the role of physiologic cyclic mechanical stretch (10% linear stretch; 0.33 Hz: light exercise conditions) on size-dependent cellular uptake and transepithelial transport of nanoparticles (100 nm) and microparticles (1,000 nm) for alveolar epithelial cells (A549) under ALI conditions. Our results show that physiologic stretch enhances cellular uptake of 100 nm nanoparticles across the epithelial cell barrier, but the barrier becomes permeable for both nano- and micron-sized particles (100 and 1,000 nm). This suggests that currently used static *in vitro* assays may underestimate cellular uptake and transbarrier transport of nanoparticles in the lung.

## Introduction

The lung is the largest organ of the human body built to accommodate the extraordinary size of required gas (oxygen-carbon dioxide) exchange surface area (ca. 100 m^2^) corresponding to about half the size of a tennis court (Weibel, 1970, 2009) within a relatively small volume (ca. 6 l; <10% of body volume). Direct exposure to airborne particles, such as cigarette smoke particles, urban dust and particles from indoor sources (e.g., cooking and laser printer) jeopardizes the fragile architecture of this organ, causing pulmonary lung diseases, such as asthma and chronic obstructive pulmonary disease (Pope et al., 2009; Hänninen et al., 2010; Schaumann et al., 2014). Out of the wide size range of inhalable particles (up to 10 μm diameter), nanoparticles (NPs) with a diameter between 100 and 300 nm have been shown to undergo epithelial-endothelial translocation, i.e., they can cross the alveolar tissue barrier into the blood circulation and from there to other organs (Kreyling et al., 2013). Moreover, ultrafine NPs (<100 nm in diameter) have received increasing attention due to their enhanced surface area per mass of particles, which has been associated with both acute and chronic lung disease (Peters et al., 1997; Schmid and Stoeger, 2016).

Improvement of the predictive power of pre-clinical *in vitro* models as an alternative to animal experiments according to the 3R principles (replacement, refinement, and reduction) relies on enhancing their biomimetic features. Over the past decades *in vitro* cell culture models of the lung epithelial cells have evolved significantly from technologically simple, non-physiologic, submerged cell culture systems to an advanced level of *in vitro* cell culture models at the air-liquid interface (ALI) (Doryab et al., 2016). In these advanced lung models, epithelial lung cells are seeded on the apical (air) side of a porous/perforated membrane, which is in contact with the cell culture medium located on the basal side. This setup mimics *in vivo* conditions, initiating polarization of cells, and secretion of protective lining fluids (surfactant), which do not occur under submerged conditions where cells are completely covered with cell culture medium (Doryab et al., 2019). Hence, ALI cell cultures provide more physiologic conditions and potentially clinically more relevant results when testing drug/toxin effects on the lung as compared to submerged cultures (Paur et al., 2011).

Moreover, *in vitro* lung models have been developed to exert cyclic mechanical stretch to cells mimicking the breathing-induced cyclic stretch conditions in the alveolar lung tissue in order to include this important stimulus for cell physiology and morphology in the cell culture models (Doryab et al., 2019). Hence, addition of this type of stimulus may prove useful for preclinical drug testing and assessment of toxin- and/or particle-induced toxicity. Most of the studies reported in the literature used commercially available cell-stretch technologies (e.g., Flexcell strain unit; Flexcell International Corp., USA) that are only suitable for submerged culture conditions (Edwards et al., 1999; Vlahakis et al., 1999; Hammerschmidt et al., 2004; Guenat and Berthiaume, 2018; Doryab et al., 2019).

Nevertheless, a variety of *in vitro* models has been developed to combine cyclic cell-stretch and ALI culture conditions for more biomimetic models of the alveolar air-blood barrier. Ideally, these advanced models enable (I) cyclic mechanical activation of the (multi-)cell cultures at the ALI, (II) basal perfusion of the culture medium, mimicking blood circulation, and (III) dose-controlled, aerosolized substance delivery. While the former two items have been implemented in various models, the latter is often missing (Doryab et al., 2019; Artzy-Schnirman et al., 2020). In 2010, the seminal work performed by Ingber et al. at the WYSS Institute of Harvard University introduced a microfluidic lung bioreactor often referred to as “lung-on-a-chip” (Huh et al., 2010). The concept of these systems is comparable to standard (multi-)cell culture models of the lung cultured at the ALI on an elastic, perforated membrane, which can be mechanically activated (stretched) combined with basal medium perfusion on a miniature-scale (shift from milli- to microfluidic system). Nowadays, these microfluidic systems have been evolved from a simple bi-channel structure (Huh et al., 2010; Stucki et al., 2015) to a complex airway network (acini-on-chips) (Artzy-Schnirman et al., 2019). However, wide-spread use of these systems is still hampered by the high degree of complexity associated with operating these systems (Ehrmann et al., 2020). These types of biomimetic alveolar barrier models not only have the potential to predict clinical outcome during early preclinical drug or toxin testing and accurate but also for mimicking drug/particle transport from the lung into the blood. In fact, the latter is part of the clinical testing (phase I of clinical trial) required for regulatory licensing of safety and efficacy of novel drugs.

All of these models and recent developments suffer from a lack of a suitable biomimetic membrane, acting as a cell-substrate. An appropriate membrane should emulate the main characteristics of the supporting extracellular matrix (ECM) of the cells, such as thickness, stiffness, permeability, and bioactivity. Commercially available polycarbonate (PC) and polyethylene terephthalate (PET) membranes are widely used in (static) ALI culture systems that do not mimic the stiffness (or rather “softness”) of the ECM in the lung. Silicone-based materials, such as poly(dimethylsiloxane) (PDMS, Sylgard 184) are generally cast for cell-stretch applications due to their suitable mechanoelastic properties (Doryab et al., 2019). Nonetheless, adsorption of proteins/growth factors to and leaching of uncured oligomers from PDMS membranes has been recognized as potential cause of adverse effects on cell physiology (Regehr et al., 2009). Recently, synthetic/natural electrospun scaffolds with a thickness range of ≈20–200 μm have been fabricated with suitable properties for lung cells using co-polymers consisting of poly(ε-caprolactone) (PCL)/star-shaped polyethylene glycols (sPEG) functionalized with biomolecules (Nishiguchi et al., 2017), poly-L-lactic acid (PLLA)/decellularized pig lung ECM (PLECM) (Young et al., 2017), and PCL/gelatin (Higuita-Castro et al., 2017). The stretchability of these scaffolds/membranes has not been determined as they were employed only under static cell culture conditions.

We have recently introduced a novel porous and elastic membrane for *in vitro* cell-stretch models of the lung cultured under ALI conditions (Doryab et al., 2020). This innovative hybrid biphasic membrane, henceforth referred to as **B**iphasic **E**lastic **T**hin for **A**ir-liquid culture conditions (BETA) membrane, was developed to optimize membrane characteristics for the two phases of cell-stretch experiments under ALI conditions, namely the initial cell seeding, attachment and growth phase under submerged cell culture conditions (phase I) followed by an ALI acclimatization and cell-stretch phase at the ALI (phase II). As these phases require distinctly different membrane properties, the BETA membrane has been designed to be biphasic. As the pores are initially filled with a wettable, water-soluble and hence sacrificial material (gelatin), the BETA membrane provides initially a non-porous and wettable enough (WCA ≤ 70°) substrate for initial cell adhesion and growth into a confluent epithelial monolayer on the apical side of the membrane (closed pores avoid inadvertent transmembrane migration of cells) (phase I). Subsequently, dissolution of the sacrificial material results in sufficient porosity, permeability and stretchability for up to 25% reversible linear strain (without plastic deformation), granting suitable ALI cell culture conditions under cyclic mechanical stretch (phase II). In contrast to typically used stretchable poly(dimethylsiloxane) (PDMS) membranes, the BETA membrane is bioactive enough to support the proliferation and formation of a confluent layer of alveolar (A549) and bronchial (16HBE14o^−^) epithelial cells without pre-coating with ECM proteins (e.g., Matrigel) (Doryab et al., 2020).

Right now, the main limitations of the BETA membrane are the relatively larger thickness (ca. 5 μm) compared to the alveolar-capillary tissue barrier (ca. 1 μm) and higher stiffness [uniaxial Young's modulus: 1.8 ± 0.7 MPa (1D stretch); 0.78 ± 0.24 MPa (3D stretch)], which is similar to or better than other typically used porous membranes for lung cell-stretch cultures (e.g., PDMS), but still about 100-fold lager than the elastic modulus (3–6 kPa) reported for alveolar walls/tissue (Doryab et al., 2020). Moreover, the ideal membrane is as bioactive as possible to provide optimum growth conditions for (primary) cell cultures and perfectly permeable to minimize membrane effects on transbarrier transport measurements.

In the present study, we attempted to improve the previously described limitations of the “optimum” BETA membrane with respect to thickness, permeability, elasticity (elastic modulus and stretchability), and bioactivity by changing its chemical composition. This is supported by newly applied analytical parameters (e.g., 3D porosity and mapping of surface topology of the membrane). Moreover, we provide a detailed technical description of the recently introduced **C**yclic ***I****n* ***VI****tro* **C**ell-stretch (CIVIC) bioreactor for cell-stretch experiments under ALI conditions (Doryab et al., 2020) with particular attention to refinements over its earlier version (MALI, **M**oving **A**ir-**L**iquid **I**nterface bioreactor) (Cei et al., 2020). Subsequently, the effect of cyclic stretch on the particokinetics of aerosol-delivered nano- (100 nm) and microparticles (1,000 nm) in an alveolar tissue barrier model (A549) cultured under ALI conditions was investigated quantitatively with respect to size-dependent cellular uptake and transepithelial transport of particles.

## Results

### Advanced *in vitro* Cell-Stretch System (CIVIC)

**Figure 1:**
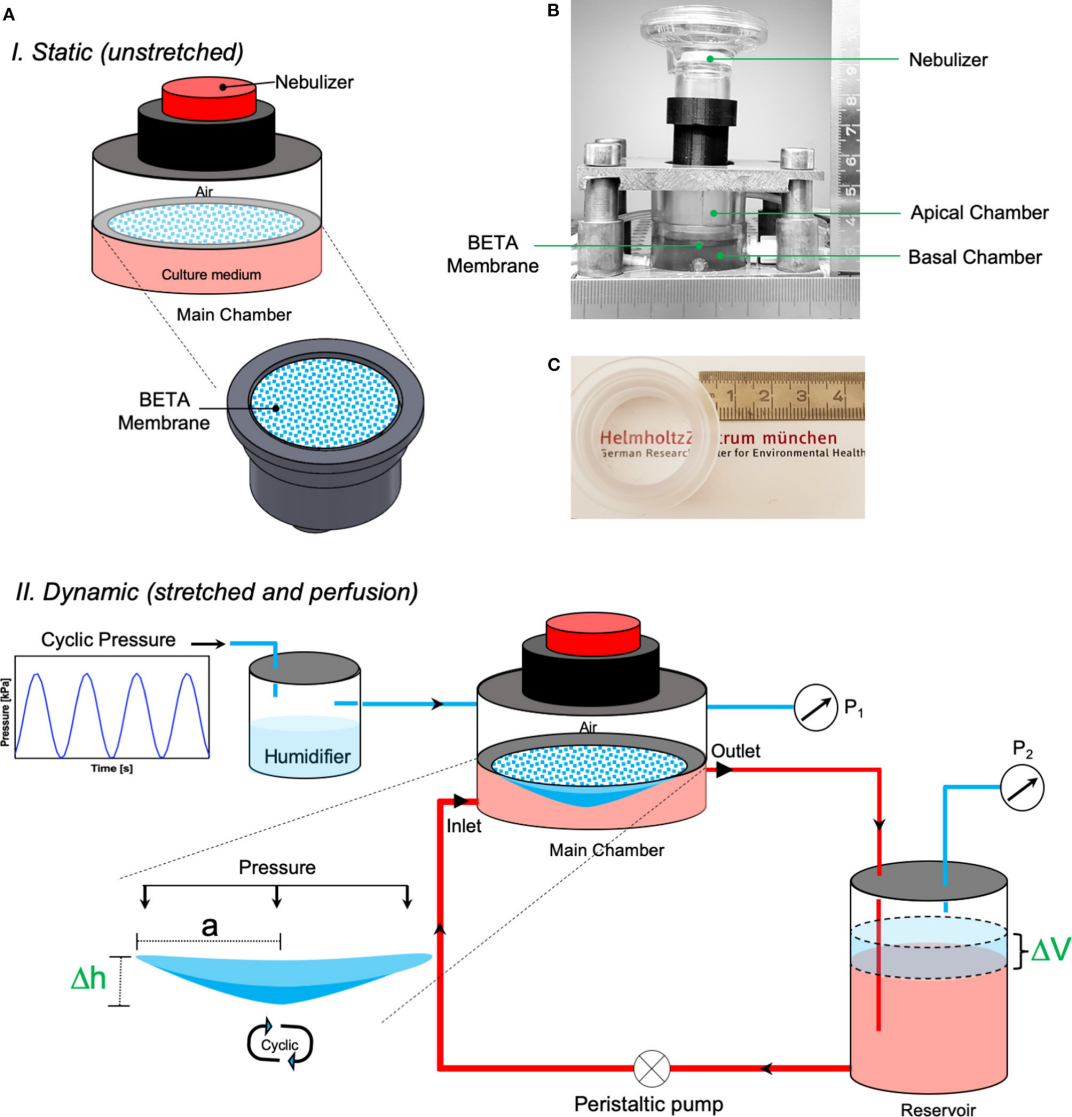
The CIVIC system used in this study. **(A)** Schematic depiction of the CIVIC system under (top) static (unstretched) and (bottom) dynamic (stretched and perfusion) conditions with the pressure-based strain/elasticity monitoring system. A thin, permeable, and stretchable membrane (BETA) placed in the (PDMS-free) main chamber of the bioreactor separates the apical (air) and basal (medium) compartments. Lung cells are grown on the membrane at ALI and perfused with culture medium by circulating the medium in the basal compartment with a perfusion pump to mimic blood flow. Cyclic mechanical stretch is applied to the cells on the membrane by applying cyclic (positive) pressure (P_1_) to the apical compartment. The cell/membrane stretch profile can be monitored via a pressure sensor in the air volume of the medium reservoir (P_2_), which is connected to the main chamber. The apical compartment of the bioreactor can be connected to a nebulizer to deliver aerosolized particles/drugs to the cells. **(B)** Snapshot of the main chamber of the CIVIC bioreactor system. **(C)** Photograph of the BETA membrane in the PC holder of the CIVIC system, which is transparent, thus favorable for direct cell imaging applications.

The **C**yclic ***I****n* ***VI****tro* **C**ell-stretch (CIVIC) system, which was employed for cell-stretch experiments with the BETA membrane, is a modified version of our previously described MALI system (Cei et al., 2020) mainly with respect to material stability, (BETA) membrane fixation, pressure sealing, and quality control including real-time monitoring of the amplitude and frequency of the cyclically stretched cell-covered membrane. The CIVIC system allows for culturing of lung epithelial cells under ALI, cyclic mechanical stretch, and medium (blood) perfusion conditions in combination with dose-controlled delivery of aerosolized substances to the cells. This *in vitro* scenario resembles closely aerosol deposition onto the air-blood barrier of the lung as encountered during inhalation therapy or breathing of ambient aerosol. The details of the technical aspects of the CIVIC system are presented in Figures 1A,B and the Methods and Materials section. A movie of the cyclically stretched membrane in the “breathing” CIVIC system can be found in the Supplementary Video 1.

### Characterization and Optimization Bioinspired Stretchable Membrane (BETA)

**Figure 2:**
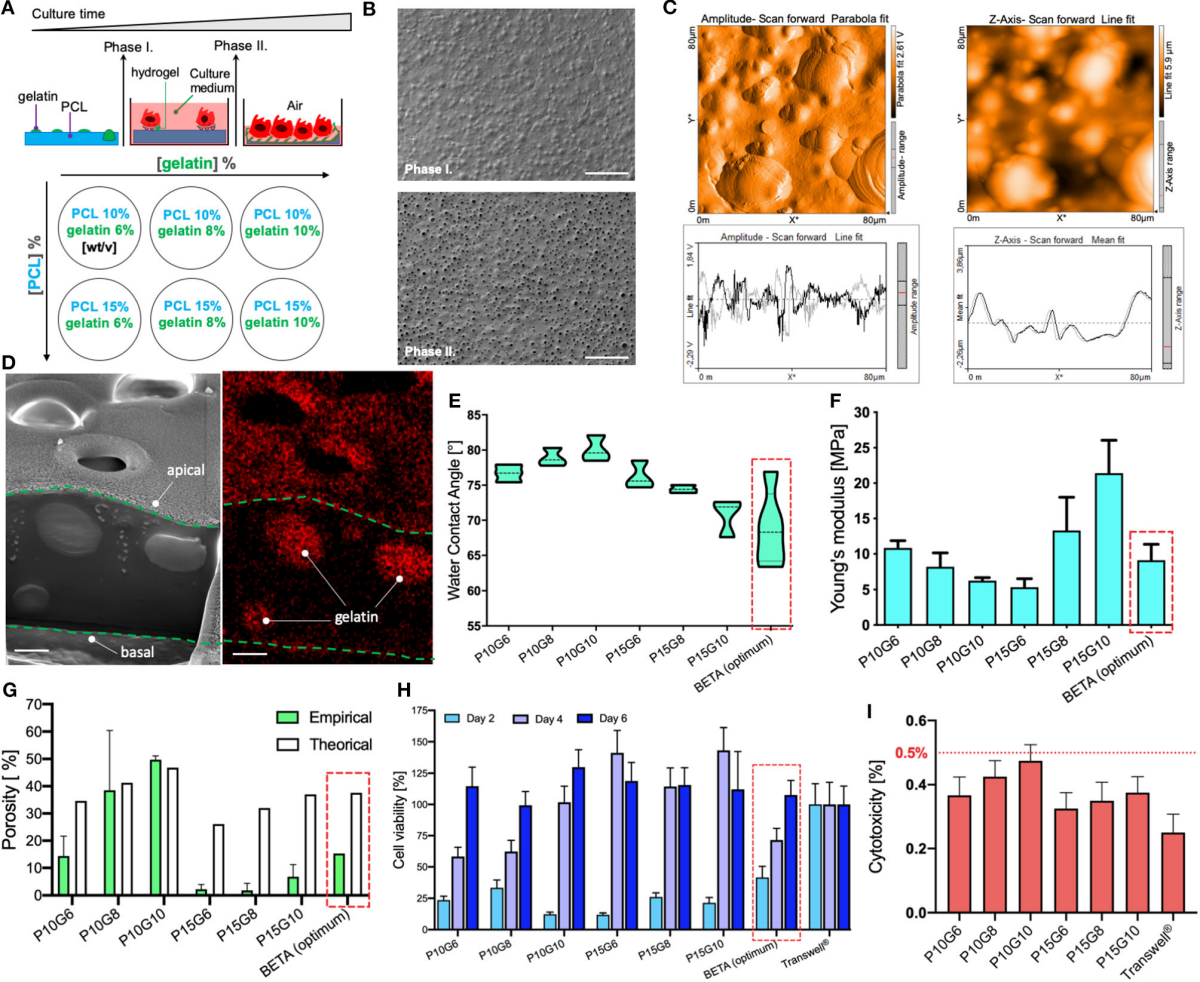
Characterization of the biphasic (BETA) membrane used in this study. **(A)** Schematic depiction of the biphasic membrane concept. (Top) During phase I, gelatin forms a hydrogel due to contact with water (cell culture medium), which serves as adhesion point for epithelial cells of the lung and facilitates subsequent cell proliferation until a confluent epithelial cell layer is formed. After 4 days (phase II), the gelatin has been dissolved in water leaving behind a network of interconnected pores in the PCL membrane, which provides space for further cell spreading and at the same time enhances both membrane permeability and elastic modulus. (Bottom) Different membranes with various combinations of mixing ratio of PCL and gelatin—in the PCL/gelatin solution used for membrane manufacturing—expected to obtain a wide range of physicomechanical properties. **(B)** Top view of the ultrastructure of the “optimum” membrane (9.35% PCL and 6.34% gelatin [w/v of TFE], i.e., P/G = 9.35/6.34), which is also used for the analysis presented in **(C,D)**. The scale bar is 100 μm. **(C)** Surface topography of an 80 × 80 μm^2^ section of the membrane analyzed by Atomic Force Microscopy (AFM; left) and its corresponding z-amplitude profile (right) showing an average roughness height of 1.31 μm. **(D)** Cross-sectional analysis of the membrane using Focused Ion Beam-Scanning Electron Microscopy (FIB-SEM, left panel) and Energy Dispersive X-ray Spectroscopy FIB-SEM (EDS-FIB-SEM, right panel), indicating that gelatin is distributed throughout the PCL membrane during the late stage of phase I. The thickness of the membrane thickness ca. 5 μm. The scale bar is 2 μm. The newly manufactured membranes (P15G6, P15G8, and P15G10) are analyzed (and compared to previously reported results (Doryab et al., 2020) with respect to **(E)** Water Contact Angle (WCA), **(F)** Young's modulus (uniaxial, phase I, under dry conditions), **(G)** porosity obtained empirically by the liquid displacement method—and theoretical (maximum) porosity (gelatin volume fraction), **(H)** cell viability analyzed by WST1 assay on days 2, 4, and 6 of culture relative to PET Transwell® cell culture insert **(H)**, and **(I)** cytotoxicity (LDH assay at day 6) of the three newly investigated membranes with different mixing ratios of PCL and gelatin. The LDH release for each membrane was normalized by the maximum possible LDH level (LDH contained in all cells). Typically, LDH < 10% is considered non-cytotoxic. There is no significant difference between the LDH release of Transwell® inserts and the different mixing ratios of BETA membranes. Optimum values for WCA, Young's modulus, empirical porosity, and theoretical (maximum) porosity were 69 ± 5 [°], 9.0 ± 1.9 [MPa], 15.32 [%] and 37.6 [%], respectively. Data are reported as the mean ± SD, *n* = 3.

As mentioned above, we recently described an optimized biphasic copolymeric membrane for cell growth inspired by the ECM in the alveolar region of the lung (Doryab et al., 2020). The initially non-porous membrane consists of two polymeric components namely poly(ε-caprolactone) (PCL) and gelatin, tailored to facilitate initial cell adhesion and growth under submerged condition (phase I) (Figures 1C, 2A). Upon contact with cell culture medium, the gelatin at the surface of the membrane turns into a hydrogel, which is conducive to cell growth (lowers water contact angle; provides favorable conditions for cell adhesion and proliferation). Gelatin also serves as “sacrificial” material, i.e., it is gradually dissolved by the medium turning the initially non-porous, stiff membrane into a porous/permeable and more elastic membrane as required for nurturing ALI cell cultures via basolateral medium and during cell-stretch experiments (phase II) (Figure 2B). We previously determined the optimum concentrations of PCL and gelatin (9.35% PCL and 6.34% gelatin [w/v solvent]) for membrane fabrication by spin coating with respect to matching the properties of the BETA membrane to the basement membrane of the alveolar tissue using a widely used) optimization approach [design of experiment (DoE)].

Due to remaining limitations of the “optimum” BETA membrane with respect to thickness and stiffness (ca. 100-fold reduction needed to match alveolar basement membrane) (Polio et al., 2018; Doryab et al., 2019; Bou Jawde et al., 2020), we attempted to improve the performance characteristics of the membrane by expanding the previously tested range of PCL/gelatin mixing ratios. For this, new membranes were manufactured with PCL concentration larger than the previously explored upper limit of 10% [w/v solvent], namely 15% PCL mixed with 6, 8, and 10% [w/v] (Figure 2A). The characteristics of these three newly generated BETA membranes were compared with previously characterized membranes consisting of 10% PCL mixed with 6, 8, and 10% [w/v] and the optimum BETA membrane (9.35% PCL and 6.34% gelatin [w/v]).

Surface analysis of the optimum BETA membrane (9.35% PCL and 6.34% gelatin [w/v solvent]) using Atomic Force Microscopy (AFM) showed an average roughness height of 1.31 μm (Figure 2C). The cross-sectional structure of the membrane was studied using Focused Ion Beam-Scanning Electron Microscopy (FIB-SEM). The data show that gelatin forms spherical “islands” in the PCL membrane (Figure 2D, left panel). These gelatin islands also extend deep into the PCL membrane, leaving a favorable interconnected 3D network of pores after dissolution of the gelatin in phase II, as confirmed by Energy Dispersive X-ray Spectroscopy (EDS)-FIB-SEM (Figure 2D, right panel).

Analysis of the water contact angle (WCA)—one of the key properties of cell attachment and growth of the three new membranes reveals a range of 70–76° indicating that all of these membranes are wettable (Figure 2E). The uniaxial tensile test of the membranes prior to cell seeding (in phase I) revealed that Young's modulus (elastic modulus) varies between 5.33 ± 1.90 and 21.41 ± 4.65 MPa (Figure 2F). Moreover, all of the membranes can endure at least 8% linear reversible strain, which is required for physiologic cell stretch conditions in the lung (Doryab et al., 2019), except for PCL 10% gelatin 6% [w/v], which can withstand only 4% linear strain. Another important parameter for culturing of cells under ALI conditions is the porosity of the membrane at the end of phase II (ALI culture), which varies between 1.8 ± 2.5 and 49.7 ± 1.4%, where the highest and lowest porosity corresponds to the membrane consisting of PCL 10% gelatin 10% and PCL 15% gelatin 8% [w/v], respectively (Figure 2G). It is noteworthy, that there is excellent agreement between empirically determined porosity and theoretically derived (upper limit of) porosity (volume fraction of gelatin), if the composition-derived theoretical porosity exceeds 40% (here: PCL 10% gelatin 8% and PCL 10% gelatin 10%).

**Figure 3:**
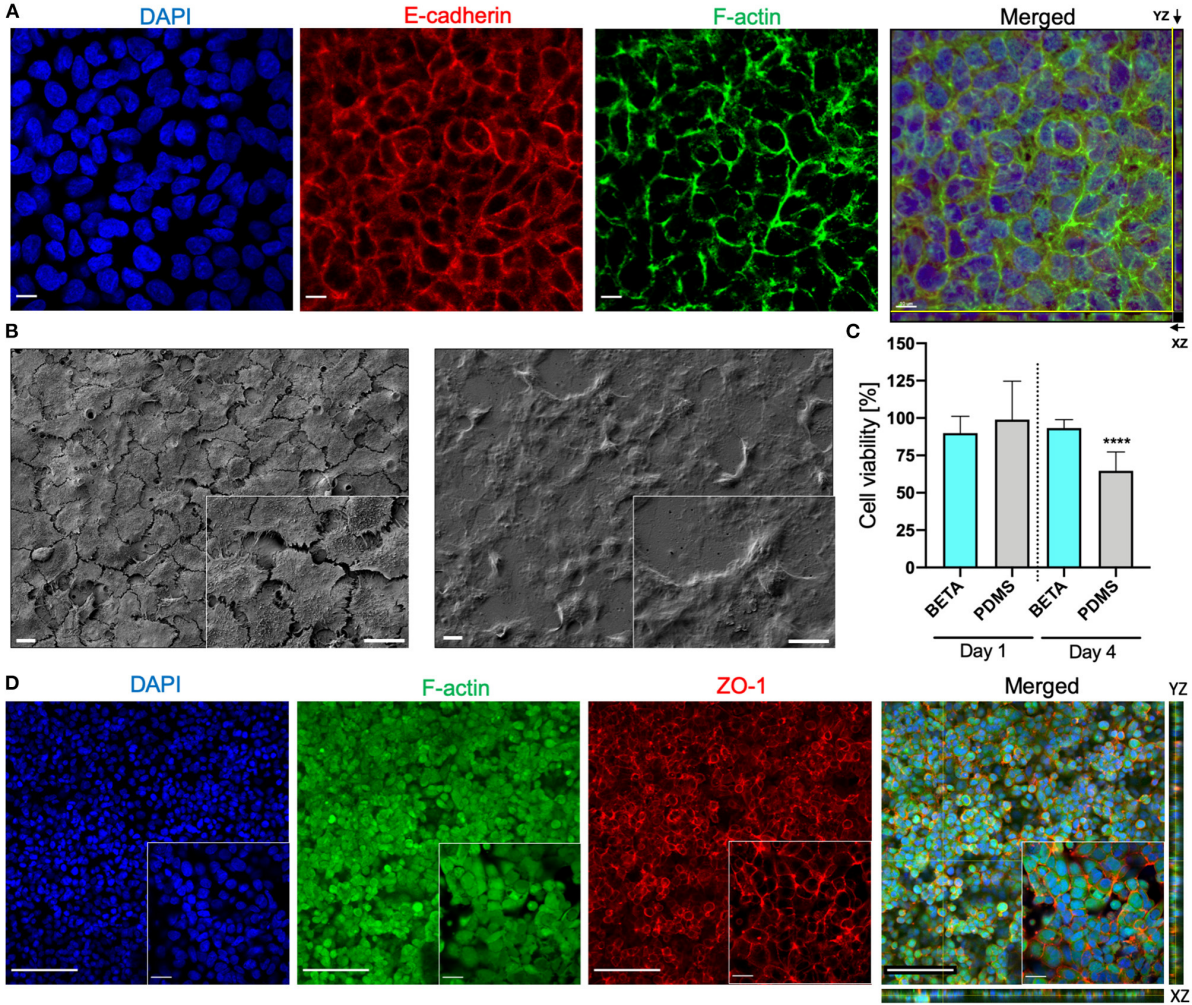
Bioactivity of the BETA membrane (optimum: 9.35% PCL and 6.34% gelatin [w/v of TFE]). **(A)** Z-stack Confocal Laser Scanning Microscopy (CLSM) of human alveolar epithelial cells (A549) on the membrane (under static submerged culture conditions for 6 days) demonstrating the formation of a confluent uniform cell layer. The cell nuclei (DAPI, blue), expression of the cell-cell adhesion protein E-cadherin (red), and formation of F-actin filaments (green). The scale bar is 10 μm. **(B)** SEM image of A549 cells after proliferation on a (left) biphasic membrane and (right) a commercial Transwell^®^ insert (6 days of submerged culture). The scale bar is 10 μm. **(C)** Effect of leaching from BETA and PDMS membrane on cell viability (WST1 assay; A549 cells). BETA and PDMS membranes were incubated for 2 days in cell culture medium and this medium was used to grow A549 cells for 1 and 4 days in a standard 12-well tissue culture well plate. The reduced cell viability for PDMS-conditioned medium after 4 days indicates that some substances (e.g., uncured oligomers) leaching from the PDMS have a cytotoxic effect. This effect is not seen for the BETA membrane. The viability data were normalized that of a standard 12- well cell culture plate with fresh medium. Data are reported as mean ± SD. *n* = 8; ^****^P < 0.00001 by one-way ANOVA with Dunnett test. **(D)** Z-stack and orthogonal CLSM view of (XY) with side views of YZ (right) and XZ (bottom) optical projection of the human bronchial epithelial cells (16HBE14o^−^) on the membrane visualizing a confluent cell monolayer and the formation of tight junctions (culture conditions: 6 days submerged and 24 h ALI culture); cell nucleus (DAPI, blue), F-actin filaments (green); ZO-1 tight junction (red). The scale bars are 100 μm (for the 20× projection view) and 20 μm (for the 63× projection view).

In addition, repeatedly performed WST1 assays showed that the metabolic activity (cell viability) of A549 cells increases with incubation time on the membranes (Figure 2H). This indicates that the relatively few initial seeded cells are proliferating and gradually covering the entire membrane as indicated by WST1 values near 100%, representing the WST1 signal obtained for standard Transwell PET inserts. After a 4–6 days growth period (depending on membrane composition), all of the membranes are covered with a confluent monolayer layer of epithelial cells (Figure 3A). As an additional measure of cytocompatibility, the release of intracellular lactate dehydrogenase (LDH) from the cytosol due to uncontrolled cell death was measured. The small release of LDH (<0.5% of totally available LDH) indicates that these membranes display low cytotoxicity (Figure 2I), which implies that the membranes do not release or leach significant amounts of toxic materials when incubated with the cell culture medium.

In summary, the three new membranes consisting of 15% [w/v] (with 6–10% [w/v] of gelatin) did not improve the key characteristics of a membrane for cell-stretch experiments at the ALI. While their wettability as quantified by WCA was not statistically different from that of the optimum BETA membrane (Figure 2D), the membrane with the lowest Young's modulus and hence highest elasticity (prior to removal of gelation) displayed an extremely low porosity of 2% as compared to the 15.32% of the optimum BETA membrane (Figure 2F). Such a low porosity value is a knock-out criterion for the membrane since it prevents efficient trans-membrane transport of nutrients (or drugs or nanoparticles) and hence nourishment of cells cultured at the ALI. Hence, the previously determined optimum BETA membrane (9.35% PCL and 6.34% gelatin [w/v solvent]) is superior to all of the 15% PCL membranes and therefore remains the composition of the optimum BETA membrane, which is used for cell experiments described below.

### Bioactivity of the BETA Membrane

We also evaluated the bioactivity of the (optimum) BETA membrane using two lung epithelial cell lines namely human lung alveolar epithelial cells (A549) and human bronchial epithelial cells (16HBE14o^−^). CLSM (confocal laser scanning microscopy) analysis showed that A549 cells grew on the membrane into a confluent cell monolayer resulted in the formation of E-cadherin—a transmembrane adhesion protein—which plays a pivotal role in cell-cell contact and polarization of cells at the ALI. Moreover, F-actin rich regions representing a network of polymeric microfilaments of the cytoskeleton were formed, which is essential for important cellular functions, such as cell motility, cell division, vesicle and organelle movement, cell signaling as well as the establishment and maintenance of cell-cell junctions and cell morphology (Figure 3A; images reflect day 6). From the DAPI-stained images (Figure 3A) the number of cells (nuclei) per surface area was determined (4.3 × 10^5^ cells cm^−2^). Since this value is 2.9-fold larger than the seeding density of the cells (1.5 × 10^5^ cells cm^−2^), this indicates that substantial cell proliferation has occurred during the 6 days of cell growth under submerged culture conditions. For reference, we also compared the bioactivity of the BETA membrane with that of a commercially available standard Transwell^®^ insert (PET) membrane. It is evident that while initial cell growth/metabolic activity (WST1 signal) on the BETA membrane was slower/lower, this difference had disappeared on day 4–6 (Figure 2H). The ultrastructural analysis exhibited a flattened cell morphology when cells grew on the BETA membrane (Figure 3B). Furthermore, cells showed superior interaction and integration with the BETA membrane as compared to the Transwell^®^ insert (Figure 3B).

PDMS (Sylgard 184) membranes are commonly used in cell-stretch devices due to their mechano-elastic properties. However, it has been reported that uncured oligomers of PDMS are released into the culture medium during cell culture, which might be toxic for the cells (Regehr et al., 2009; Carter et al., 2020). Hence, we assessed the leaching of unwanted components of the PDMS membrane compared to the (optimum) BETA membrane (Figure 3C). Medium incubated for 48 h with PDMS and BETA membranes was used to culture A549 cells under submerged conditions. After 1 day, no significant difference was detected in cell viability for the two materials. However, after 4 days of incubation, a 36% reduction in cell viability was detected for the cells incubated with PDMS-leached medium, while only 6% of viability reduction was observed for the BETA membrane-leached medium as compared to cells cultured with pristine cell culture medium.

We also examined the bioactivity of the BETA membrane using the human bronchial epithelial cell line 16HBE14o^−^. Cells were grown on the (optimum) BETA membrane for 6 days under submerged and 1 day under ALI conditions. A confluent epithelial barrier with the formation of the F-actin was observed (Figure 3D). SEM analysis also confirmed the CLSM findings (Supplementary Figure 2). Excellent barrier integrity was also confirmed by a TEER value of 451 ± 55 Ω cm^2^, which is consistent with data reported for 16HBE14o^−^ cells in the literature (Ehrhardt et al., 2002) and higher than the TEER value for A549 epithelial confluent cell monolayer on the BETA (136 ± 23 Ω cm^2^).

### Nano- and Microparticle Kinetics Study

As an application of the *in vitro* cell-stretch lung model (CIVIC), we investigated the cellular uptake by and transepithelial transport of nano- and microparticles of A549 cells under physiologic cyclic mechanical stretch (10% linear, 0.33 Hz) applied for 2 h. The stability of the stretch parameters as well as the stiffness [Young's modulus: 0.78 ± 0.24 MPa (mean ± SD, *n* = 5)] of the membrane was monitored continuously during the entire experiment with the differential pressure monitoring system of the CIVIC system. The metabolic activity of the cells did not show any evidence of reduced viability due to aerosol exposure and 2 h of cyclic stretch (Supplementary Figure 3).

**Figure 4:**
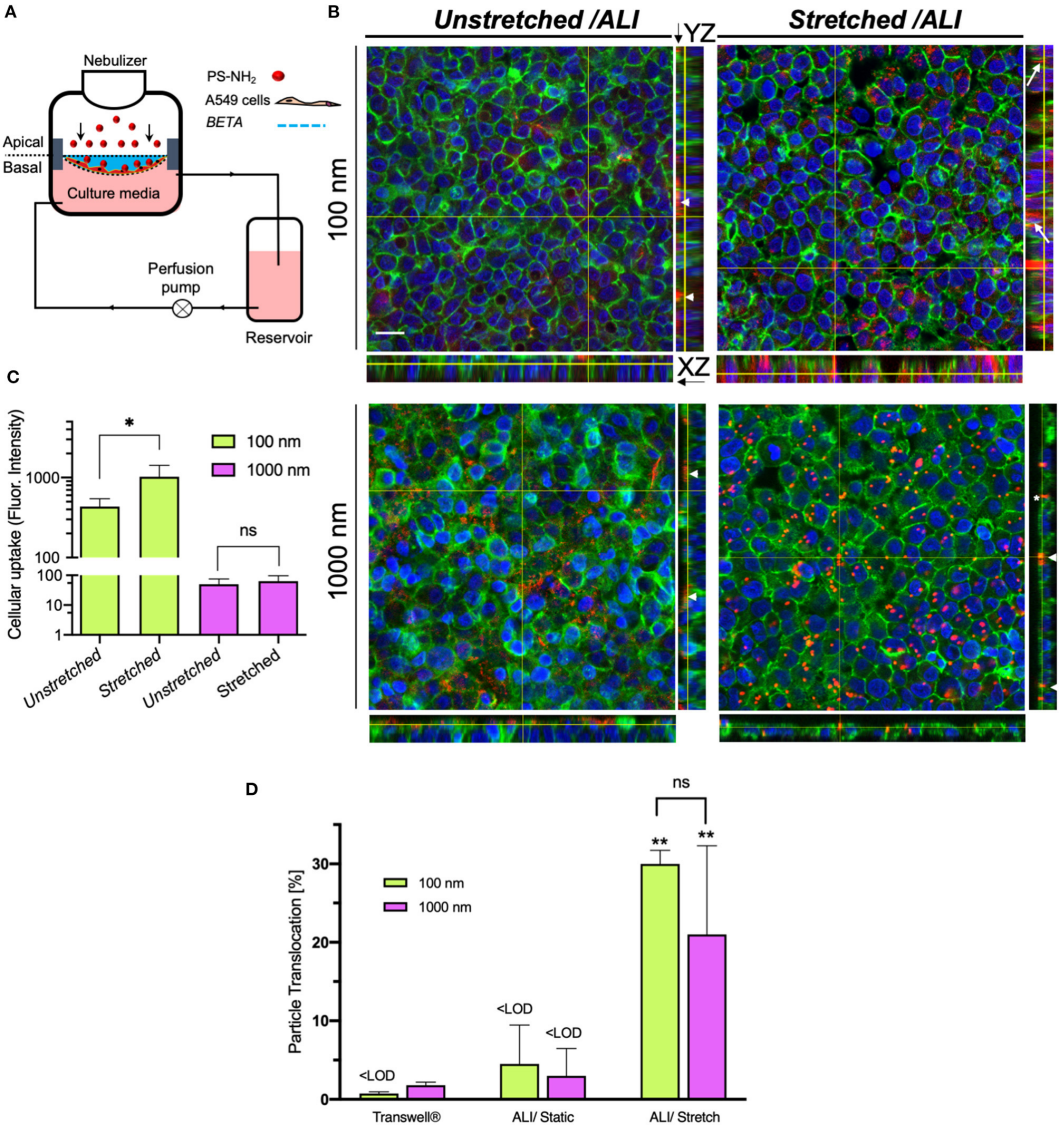
Cellular uptake and membrane-association of aerosolized nano- and microparticles for alveolar epithelial (A549) cells and translocation across the epithelial barrier (2 h incubation). **(A)** Schematic of the CIVIC bioreactor system for particle study. A549 cells were seeded on the BETA membrane (cell density: 2 × 10^5^ cells cm^−2^, 4 days LLC, and 1-day ALI culture). Amine-modified polystyrene (PS-NH_2_) nano- and microparticles (100 and 1,000 nm diameter, respectively) are then nebulized onto the cells with the nebulizer of the bioreactor. After 2 h, the cells were fixed and prepared for CLSM analysis. **(B)** 3D reconstruction z-stack of CLSM images presented as orthogonal (XY) and side views (YZ, right) of monolayered, confluent cells on the membrane after nebulization of 100 and 1,000 nm fluorescently labeled, amine-modified polystyrene (PS-NH_2_) particles under non-stretched and physiologically stretched (10% linear, 0.33 Hz for 2 h) under ALI conditions. Cell nucleus (DAPI, blue), particles (red) and F-actin filaments of the cytoskeleton (green). Arrows, arrowheads, and asterisk indicate internalized particles, cell-membrane associated (extracellular) particles (on the apical cell surface) and particles located between cells, respectively. (Scale bar: 20 μm). **(C)** Quantitative cellular uptake of particles measured by fluorescence intensity of z-stacks, showing that physiologic cyclic mechanical stretch enhances uptake of 100 nm NPs as compared to static conditions, while there is no effect on 1,000 nm microparticles [Representative images (z-stacks) were recorded at 5 independent fields of view for each sample (*n* = 4); region of interest: 134.95 × 134.95 μm]. Y-axis is presented fluorescence intensity data in a log scale. Data are reported as the mean ± SD; ^*^P < 0.01 by two-way ANOVA and data were corrected by Sidac for multiple comparison tests. **(D)** Translocation of 100 and 1,000 nm particles across the cell layer grown on unstretched PET Transwell^®^ inserts and on the BETA membrane (under unstretched and stretched conditions) (*n* = 3). ^**^ Show the comparison between stretched with the corresponding experiment under unstretched conditions. Data are reported as the mean ± SD; ^**^P < 0.001 by two-way ANOVA and data were corrected by Tukey for multiple comparison tests.

This study shows that 2 h of cyclic stretch affects cellular uptake and intracellular distribution, but not trans-cellular transport in a size-dependent way. The former is qualitatively evident from CLSM images revealing that cellular uptake of 100 and 1,000 nm particles under unstretched (static) ALI conditions was very limited and cell-associated particles were mostly located close to the air-facing, apical cell surface (Figure 4B). In contrast, 100 nm NPs were internalized more efficiently under stretch conditions and co-localized with the F-actin cytoskeleton deeper within the cell (Figure 4B). On the other hand, stretch did not enhance cellular uptake of 1,000 nm microparticles and particles were still localized close to the apical cell surface, but positioned preferably between adjacent cells rather than randomly as without stretch (Figure 4B). Quantitative fluorescence analysis of the CLSM images revealed a 2.4-fold increase of cellular uptake of 100 nm NPs under stretch, while there was no statistically significant effect of stretch on cellular uptake for 1,000 nm microparticles (Figure 4C).

In addition, spectrophotometric analysis of the basal medium revealed the transepithelial transport (translocation) of particles after 2 h under static and stretch-activated conditions of A549 cells on the BETA membrane or on 3 μm pore (static) PET membranes of 6-well Transwell^®^ inserts (Figure 4D). For Transwell^®^ inserts, the particles were delivered with a VITROCELL^®^Cloud 6 system to A549 cells (Lenz et al., 2014) and then cultured under (static) ALI conditions.

After 2 h of incubation time under static conditions, the transport fractions across A549 cells on both Transwell^®^ inserts and BETA membranes for 100 and 1,000 nm particles were below the detection limit, except for the 1.8 ± 0.4% transport of 1,000 nm particles observed for Transwell^®^ inserts (two-way ANOVA followed by *post-hoc* Tukey's multiple comparison test) (Figure 4D and Table 1).

**Table 1:** Transport of 100 and 1,000 nm particles across A549 cell-layer grown on BETA or PET Transwell^®^ insert membrane (3 μm pores) at ALI within 2 h of particle exposure (mean ± SD; *n* = 3).

** 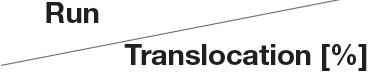 **	**100 nm**	**1,000 nm**
Transwell^®^/Unstretched	< LOD^*^	1.8 ± 0.4
BETA/Unstretched	< LOD	< LOD
BETA/Stretched	30.0 ± 1.7	21.0 ± 11.3

* *<LOD, below limit of detection*.

For cell-stretch, the transepithelial transport of 100 and 1,000 nm particles across the A549 cell-covered BETA membrane was increased to 30.0 ± 1.7 and 21.0 ± 11.3%, respectively, but no statistically significant dependence on size was observed (no stretch can be applied to Transwell^®^ inserts). Hence, cell-stretch significantly increased the translocation of 100 and 1,000 nm particles across the alveolar epithelial barrier independent of particle diameter (see Table 1 and Figure 4D).

## Discussion

In the quest for overcoming limitations of traditional *in vitro* models of the lung, the field of bioengineering has witnessed significant efforts toward developing advanced *in vitro* models striving to mimic more closely the human pulmonary environment (de Souza Carvalho et al., 2014). This has led the way from mono-cellular submerged cell lines to primary co-culture cell models at the ALI (air-blood barrier), from static cell culture media and cell layers to medium perfusion (pulmonary blood flow) and cyclic stretch (breathing-induced mechanical tissue strain), and from millifluidic (~cm^2^ cell area, mL of media) into microfluidic systems often referred to as lung/acinar-on-a-chip technologies (Huh et al., 2012, 2013; de Souza Carvalho et al., 2014; Benam et al., 2015; Tenenbaum-Katan et al., 2018; Ainslie et al., 2019; Artzy-Schnirman et al., 2019). While lung-on-a-chip technologies are starting to become commercially available (e.g., Alveolix, Switzerland and Emulate, USA), also millifluidic lung bioreactors are expected to continue to play a role due to their ease-of-handling, a larger amount of cell samples suitable for many standard assay kits, and lower maintenance efforts.

At the core of any cell-stretch lung bioreactor/chip is a porous and elastic membrane on which the cell culture model is cultured. For lung/acinar-on-a-chip systems mainly 3.5–10 μm thick PDMS membranes are used for cell seeding and growth of an alveolar or bronchial tissue barrier (Huh et al., 2010; Stucki et al., 2015). PDMS membranes are widely used for their high mechano-elasticity, with Young's modulus of ≈1–3 MPa (Wang et al., 2014). While perforated PDMS membranes are suitable for small-sized lung-on-chip applications (~mm^2^), they are too fragile for larger millifluidic (~cm^2^) devices. Moreover, PDMS membranes have low wettability (WCA ≥ 115°) (Supplementary Figure 1) and therefore require pre-treatment and/or coating with ECM proteins to facilitate sufficient cell adhesion and proliferation (Wang et al., 2010). Another disadvantage is that uncured oligomers of PDMS can leach into the cell culture medium resulting in changes and cell physiology (Regehr et al., 2009; Carter et al., 2020). Our investigation of a PDMS film has confirmed reports from the literature that cells may experience reduced cell viability due to the leaching of toxins into the cultured in a medium (Regehr et al., 2009). Alternatively, commercial electrospun biocompatible poly(carbonate)urethane (PCU) membranes (Bionate^®^ II 80A, The Electrospinning Company, UK) have been tested for cell growth in a millifluidic lung bioreactor. They proved inadequate due to their hydrophobic nature (in spite of pre-coating with ECM proteins) and associated poor cell proliferation, their relatively large thickness (ca. 75 μm), and their inability to prevent the formation of multilayered epithelial tissue deep within the membrane rather than at its apical side (Cei et al., 2020).

**Figure 5:**
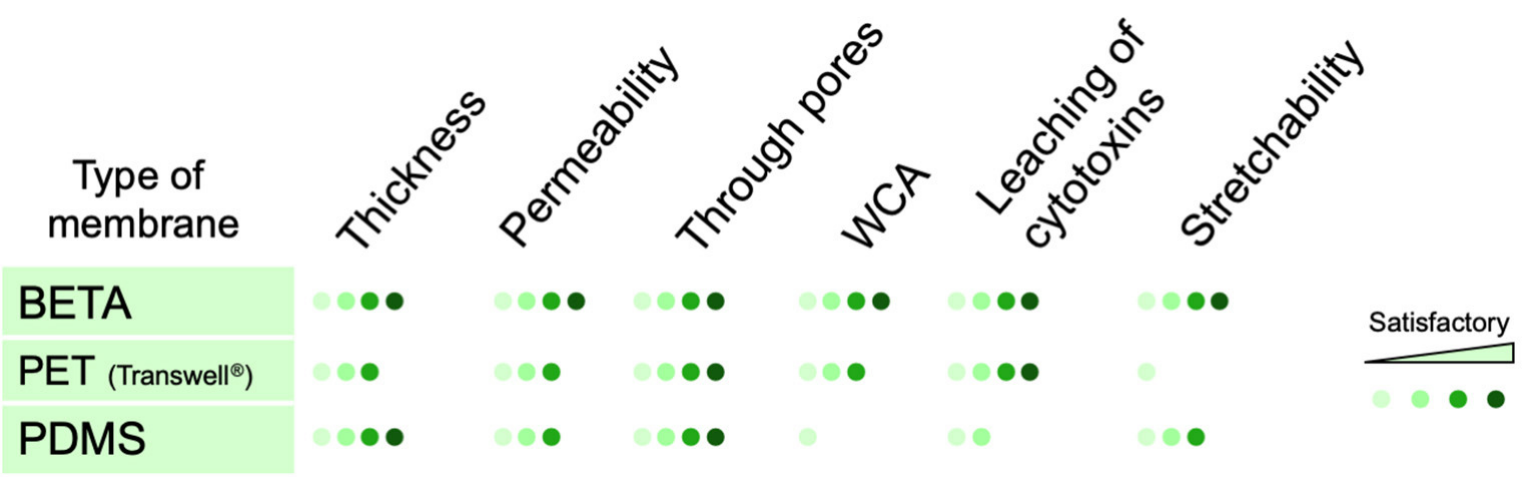
Qualitative comparison between key properties of conventional porous membranes (PET and PDMS) used for static and dynamic (cell-stretch) *in vitro* lung models at the air-liquid interface and the BETA membrane presented here. The scores are based on biomimetic relevance as compared to the alveolar basement membrane. The permeability was measured as apparent permeability (Papp) for FITC-dextran (4 kDa). Stretchability and through pores refer to the elastic modulus and pores connecting the apical and basal sides of the membrane, respectively.

The BETA membrane, which overcomes some of these limitations (Figure 5), has a thickness of ≤ 5 μm, which is thinner than conventional PET or PC/PET membranes used in static Transwell^®^ inserts (≈10 μm) and similar to the lower range of advanced PDMS membranes (≈3.5–10 μm) (Huh et al., 2010; Stucki et al., 2015). The two polymer components, i.e., gelatin and PCL were chosen for their wettability and mechanical properties, respectively. The presence of gelatin initial non-porous membrane (BETA in phase I) is conducive to cell adhesion/growth without requiring further surface modification and prevents apically seeded epithelial cells from unwanted migration through the membrane to the basal side, fostering the formation of a monolayer of epithelial cells on the apical side. The gradual dissolution of gelatin by cell culture medium induces sufficient porosity for culturing of cells at the ALI and even results in the secretion of innate ECM secreted by the cells. In contrast to PDMS, no adverse effect on cell viability due to leaching has been observed (Figure 3C).

As mentioned above, one of the main advantages of the PDMS membranes is their high mechano-elasticity with Young's modulus of ≈1–3 MPa (Wang et al., 2014), which is still ca. 100-fold larger than that of alveolar tissue with 3–6 kPa (Polio et al., 2018; Bou Jawde et al., 2020). Since the previously derived optimized BETA membrane (PCL/gelatin = 9.35%/6.34% [w/v solvent] or P/G = 9.35/6.34) (Doryab et al., 2020) has an initial Young's modulus of 9.0 ± 1.9 MPa (prior dissolution of gelatin), the present study tested the hypothesis that an increased PCL concentration of 15% (rather than 6–10% as tested previously) will result in a more elastic membrane. It became evident, that while a ca. 2-fold lower (uniaxial) Young's modulus (5.3 ± 1.2 MPa for P15G6) could be obtained (Figure 2F), the porosity would be prohibitively low (2% for P15G6) for sufficient trans-membrane nutrient transport during ALI culture conditions. Hence, the previously determined optimum BETA membrane (P/G = 9.35/6.34) was used for the cell-stretch cell experiments.

It is important to note that albeit the (optimum) BETA membrane has an initial (uniaxial) Young's modulus of 9.0 ± 1.9 MPa prior to the dissolution of gelatin (prior to phase I), the (uniaxial) Young's modulus reduces to 1.84 ± 0.66 MPa after dissolving sacrificial gelatin (day 6 under submerged conditions; end of phase II) (Doryab et al., 2020). When measured under more realistic, triaxial stretch conditions in the CIVIC (pressure monitoring method), the elastic modulus of the BETA membrane decreased from 1.33 ± 0.14 MPa (day 1; partial gelatin dissolution) to 0.78 ± 0.24 MPa (day 6) (Doryab et al., 2020), which is ca. 2-fold lower than the corresponding uniaxial value (1.84 ± 0.66 MPa). Considering that the latter was measured under dry conditions these two values can be considered equal within expected experimental uncertainties, which indicates that the BETA membrane is quite isotropic.

In the present study, we recognized the limited value of 2D porosity (pore-area fraction at the surface of the membrane), did not correlate well with the gelatin volume fraction of the membrane, for membrane optimization with respect to 3D porosity (through pores) as only an interconnected 3D pore structure allows for sufficient contact between apically located cells and basal medium during ALI cell culturing. The measurement method for 3D porosity described here was in excellent agreement with the theoretically predicted porosity from gelatin volume fraction, if the latter was larger than 41% (Figure 2G). This indicates that for gelatin volume factions larger than 41% all of the available gelatin can eventually be reached and hence dissolved by culture medium, i.e., the gelatin-induced 3D pore structure is perfectly interconnected, which is optimum for ALI culture conditions. For gelatin factions below ca. 35%, the 3D porosity falls below 10% implying that increasing “islands” of gelatin are formed, which are completely engulfed by non-soluble PCL (Figure 2B of Doryab et al., 2020). Thus, future efforts for improved membrane composition should focus on gelatin volume fractions near or above 40% to provide sufficient 3D porosity.

In summary, the optimum BETA membrane is relatively thin (≤ 5 μm) with a suitable permeability (9.9 × 10^−6^ cm s^−1^ for FITC-dextran 4 kDa and 15.3% 3D porosity), which can provide sufficient contact between ALI cultured cells on the apical and medium on the basal side of the membrane (Weibel, 1970; Doryab et al., 2019). The BETA membrane is also stretchable up to 25% linear strain (during phase II), which includes the range of physiologic strain (up to 12% linear) and non-physiologic over-stretch conditions as discussed below. While the BETA membrane is more biomimetic than most other membranes currently available due to the low WCA (68°) and 3D interconnectivity of the pores, the BETA membrane still has a ca. 100-fold higher elastic modulus and thickness of the alveolar tissue and basement membrane, respectively (Polio et al., 2018; Doryab et al., 2019; Bou Jawde et al., 2020). The former is expected to alter cell physiology as compared to lung conditions and the latter may result in bias transbarrier transport studies. Future research on membrane technology mimicking the alveolar basement membrane should focus not only on matching the physicochemical properties of the basement membrane but also on its microstructural (network of ECM fibers) and molecular structure.

Thus, further research is needed to close this physiologic gap. Several alternative synthetic scaffolds have presented promising results for lung, such as biofunctionalized or synthetic-peptide-based synthetic scaffold (Nishiguchi et al., 2017). Despite the natural-based scaffold (e.g., collagen type I), synthetic scaffolds can be tailored to have selective tunable properties, mimicking the microenvironment of cells to facilitate cell adherence, proliferation, and differentiation. However, artificial scaffolds, i.e., natural, synthetic, and natural/synthetic introduced until now are unable to concurrently mimic all the physical, mechanical, and biological properties of the natural ECM or basement membrane of the pulmonary cells. The biomimetic biphasic scaffold reported here has excellent structural, mechanical, and biophysical characteristics and can be a suitable alternative for growing epithelial cells not only in the monoculture but also in co-culture and triple co-culture models (Lehmann et al., 2011; Heydarian et al., 2019).

Ultimately, sufficiently biomimetic *in vitro* models of the alveolar tissue should not only focus on the basement membrane but also include more advanced primary alveolar cell models, such as the commercially available primary-derived hAELVi cells (Kuehn et al., 2016) or EpiAlveolar cell model (MatTek, Inc.) (Barosova et al., 2020). The combination of both aspects should lead to even more biomimetic and hence clinically predictive models of the alveolar barrier.

The millifluidic CIVIC bioreactor utilizes positive pressure to mechanically stretch a cell-covered elastic membrane activate stretchable and thus allows for ALI cell culture conditions with medium perfusion, cyclic stretch, and monitoring of the pressures in the apical and basal compartment of the bioreactor is leveraged for real-time monitoring of amplitude and frequency of cyclic stretch and of tri-axial Young's modulus of the cell-covered membrane. This allows for precise selection of stretch conditions and quality control of key experimental conditions during the course of the cell-stretch experiment. Moreover, an aerosol-cell delivery unit using a clinically relevant nebulizer enhances its applicability to drug testing suitable for inhalation therapy.

It is instructive to relate the characteristic stretch and perfusion parameters of the CIVIC equipped with the BETA membrane to clinical conditions. The BETA membrane can sustain linear stretch amplitudes of up to 25% (Doryab et al., 2020). Population-based averages for breathing frequency and tidal volume during heavy exercise are 26 breaths per minute (33 bpm for women) and 1.92 L (1.36 L), respectively (ICRP, 1994). For typical lung inflation of 3.3 L (2.7 L) at functional residual capacity (FRC, at end of exhalation), this tidal volume corresponds to a 58% (50%) increase in lung volume yielding a 17% (15%) linear and 36% (31%) area change (assuming the alveolar sacs are spherical). Analogous, the stretch conditions were chosen here for the particokinetics study (10% linear at 0.33 Hz = 20 bpm) correspond to a tidal volume of 1.09 L (female 0.89 L), which is similar to “light exercise” conditions (male: 1.25 L, 20 bpm; female: 0.99 L, 21 bpm) (ICRP, 1994). Consequently, linear strain larger than 17% (or 35% ΔSA), which may occur for instance during positive pressure ventilation, is considered over-distension or non-physiologic strain, possibly provoking apoptosis and necrosis, increase permeability and release of inflammatory mediators (such as Interleukin-8) in alveolar epithelial cells (Edwards et al., 1999; Vlahakis et al., 1999; Hammerschmidt et al., 2004).

The maximum positive differential pressure applied to the apical side of the cells during a cyclic stretch in the CIVIC (2.5 kPa) is higher than exerted onto the lung tissue during normal exhalation (0.1–0.5 kPa), but similar to conditions during mechanical ventilation at the intensive care unit of a hospital (normal: 1.5–2.0 kPa, 2.5 kPa acceptable peak value; 3.0 kPa should not be exceeded for an extended period of time) (Hall, 2016). Finally, the medium perfusion rate of 400 μL min^−1^ has to be put into context with the area of gas exchange (membrane area: 5 cm^2^) for comparison with the lung. The ratio of area and perfusion rate of the CIVIC is with 1.25 m^2^/(L min^−1^)^−1^ considerably lower than that of the lung [20 m^2^/(L min^−1^)^−1^ = 100 m^−2^/(5 L min^−1^)], but the corresponding flow rate of 6.4 L min^−1^ in the CIVIC system technically not be difficult to establish since it would require tubing with much larger diameter enhancing the laboratory footprint unduly.

It is well known that the size of particles plays a key role in cellular uptake and internalization of the particles (Takenaka et al., 2012; Zhu et al., 2013). We found that both 100 and 1,000 nm amine-coated particles could not be internalized by alveolar epithelial cells (A549) under static/unstretched conditions (2 h after aerosolized particle delivery). Moreover, 1,000 nm microparticles were also not taken up by cells under stretched conditions, which can be explained by endocytic uptake (most relevant uptake mechanism for epithelial cells) being limited to ca. 500 nm particles (Winnik and Maysinger, 2013; Zhu et al., 2013; Annika Mareike Gramatke, 2014). In contrast, the relatively efficient cellular uptake of 100 nm NPs is consistent with previous observations that positively charged (amine-coated) NPs tend to interact with the negatively charged cell membrane, which enhances their uptake as compared to neutral or even negatively charged particles of the same size (Rothen-Rutishauser et al., 2006). The observed colocalization of NPs with the F-actin cytoskeleton in A549 cells is conducive for further intracellular trafficking and endocytosis of the NPs within cells (Foroozandeh and Aziz, 2018). For *in vitro* pharmacokinetic testing, ALI culture conditions have several advantages over submerged cell culture settings. In addition to more physiologic and tighter barrier function, burst-like pharmacokinetic profiles as typically seen in patients can only be observed for ALI conditions (Meindl et al., 2015; Schmid et al., 2017) since this resembles rapid depletion of the pulmonary drug reservoir due to drug transport into the blood. Moreover, the cell-delivered dose is often poorly known under submerged conditions due to variable particle-cell transport rates in cell culture medium (Teeguarden et al., 2007; Schmid and Cassee, 2017). In contrast, under ALI conditions, the aerosol dose is delivered immediately directly onto the epithelial barrier mimicking the conditions of inhaled particles in the lung. To date, *in vitro* particokinetic studies are mainly performed under submerged culture conditions, and to the best of our knowledge, no quantitative transbarrier particokinetic (translocation) studies under cell-stretch ALI conditions have been reported, yet.

For sub-100 nm NPs the translocated fraction of particles is inversely proportional to particle size in both static *in vitro* cell and *in vivo* animal models of the lung (Kreyling et al., 2014; Bachler et al., 2015). For A549 cells at ALI and rodent models after NP instillation, the first plateau of particle translocation is achieved within 2 h for 80 nm gold NPs, but it is considerably larger for (static) A549 cells (ca. 2% of the delivered dose) as compared to particle instillation in rats (ca. 0.2%) (Kreyling et al., 2014; Bachler et al., 2015). For A549 cells, receiving doses larger than 0.1 μg cm^−2^ for gold NPs has resulted in a decrease in the transport rate (Bachler et al., 2015).

The 2 h translocation fraction of 100 nm amine-modified polystyrene particles observed in this study was below the detection limit [ca. 1.0% (Transwell) and ca. 3% BETA], which is in general agreement well with the results from Bachler and colleagues (Bachler et al., 2015), if we consider that the larger than 0.1 μg cm^−2^ (here 2.1 μg cm^−2^) cell-delivered dose may have lowered the transport fraction. On the other hand, the 1.8% of translocation fraction for 1,000 nm particles (Transwell inserts) appears relatively large considering that virtually no translocation of microparticles has been reported in *in vivo* biokinetics studies. The absence of 1,000 nm translocation for the BETA membrane (below detection limit of ca. 3%) is consistent with these *in vivo* results. While it may be expected that cyclic stretch affects the transepithelial mobility of particles by, e.g., additional convective particle transport, changes in paracellular barrier integrity, and effects on cellular uptake and transport mechanisms, the relatively large increase to 30 and 20% for 100 and 1,000 nm particles is unexpectedly high. Since these values even agree within experimental uncertainty it is unlikely that this is the result of an active cellular transport process especially since endo-/exocytosis as the most effective cellular uptake and transport mechanism for epithelial cells is limited to sizes below 500 nm. Moreover, substantially enhanced cellular uptake was only observed for 100 nm particles (Figure 4B). Therefore, we assume that this large increase in translocation fraction is due to a combination of passive mechanisms, such as rupture of the relatively weak tight junctions of A549 cells yielding intracellular gaps, which allows for enhanced convective transport of particles irrespective of particle size due to leakage of the medium in and out of the apical space where the particles are residing.

## Conclusion

The “optimum” copolymeric BETA membrane (P/G = 9.35/6.34) applied here is biomimetic in the sense that it is thin (≤ 5 μm), surface wettable, permeable with proper pore size for cell growth and interconnected 3D pore structure, elastic and bioactive, and somewhat comparable to the ECM in the alveolar region. However, it still is ca. 100-fold too thick and stiff as compared to the basement membrane of the alveolar region. These limitations could not be alleviated by enhancing the poly(ε-)caprolactone (PCL) concentration 15% (w/v). Using the “optimum” we showed that the CIVIC can be utilized for cellular uptake and transepithelial transport studies under physiologic stretch and ALI conditions including aerosolized substance delivery. While the results for static conditions are in general agreement with literature data, unexpectedly high translocation of both 100 and 1,000 nm particles under physiologic stretch (light exercise) was observed for an A549 alveolar lung barrier. This suggests that more appropriate cell (co-)culture models with more pronounced tight junctions and advanced primary cell culture models should be employed for cell-stretch experiments. Studies in the field of respiratory diseases are expected to benefit greatly from the development of more biomimetic and reliable *in vitro* models of the lung as currently available. We believe our system presents a valuable step toward improvement of the predictive value of advanced lung cell models.

## Materials and Methods

### Cell Culture

Human alveolar type-II like epithelial cells (A549) were cultured and maintained in Dulbecco's Modified Eagle Medium: Nutrient Mixture F-12 (DMEM/F12, 1:1 v/v, Gibco) supplemented with 10% FCS (Gibco), 1% (v/v) Pen/Strep (100 U mL^−1^, Gibco), 1% L-glutamine (2 mM, Gibco), and 2-phospho-L-ascorbic acid (0.1 mM, Sigma). Human bronchial epithelial cells (16HBE14o^−^) were cultured in MEM/F12 medium (Gibco) supplemented with 10% FCS (Gibco), 1% (v/v) Pen/Strep (100 U mL^−1^, Gibco) and 1% L-glutamine (2 mM, Gibco).

For longitudinal monitoring of cell viability (WST1), A549 cells were grown on the BETA membranes by seeding cells with a cell density of 1.5 × 10^5^ cells cm^−2^ on a UV sterilized BETA membrane (effective growth area: 1.3 cm^2^, depending on application). A PTFE holder was used for keeping the BETA membranes) and on Corning^®^ Costar^®^ Transwell^®^ cell culture inserts (PET, 12-well, 1.1 cm^2^; 3 μm pore) (control). Cells were then cultured for 6 days under submerged conditions (basal and apical medium volumes were 1.5 and 0.5 mL, respectively) followed by 24 h under ALI conditions. Similarly, 16HBE14o^−^ cells were also grown on the BETA membrane (cell seeding density: 2 × 10^5^ cells cm^−2^; effective growth area: 1.3 cm^2^) for 6 days under submerged and 24 h under ALI conditions.

Immediately prior to cell-stretch experiments the membrane was placed in the CIVIC and the media volume in the basal and reservoir chamber of the CIVIC were 4 and 12 mL (including 2 mL in the connecting tubing), respectively.

### *In vitro* Cell-Stretch System (CIVIC)

We used the CIVIC system to apply cyclic stretch to cells grown on the BETA membrane under ALI culture conditions (Figure 1). The main chamber of the CIVIC bioreactor is separated by the BETA membrane into an apical (humidified air) and a basal (perfused cell culture medium) compartment mimicking the air-blood barrier of the lung including its breathing-related cyclic stretch induced by oscillation of the apical pressure (Figures 1A,B and Supplementary Video 1). The cell culture medium in the basal compartment is circulated using a peristaltic pump to mimic blood flow (400 μL min^−1^). Cells grown on the membrane are subjected to a uniform cyclic triaxial strain by applying a cyclic (here: sinusoidal) positive pressure to the apical chamber by cyclic opening of a valve connected to pressurized cleaned house air and a valve connected to ambient air. The entire system is placed in an incubator (37°C) to maintain optimum cell culture conditions. The dry house air is entering the chamber for pressurization via a humidifier and the initially dry air in the apical compartment can be humidified by nebulization of small volume (2 μL) of saline using the nebulizer described below (Figures 1A,B). Both amplitude and frequency of stretch can be set by an Arduino integrated development environment (or IDE) software (Arduino IDE 1.0.5 for Windows). For BETA membranes, the CIVIC bioreactor is able to apply physiologic linear strain: (0–17%) and non-physiologic (over-stretch; 17–25%) stretch conditions, as described below.

The CIVIC system is a modified version of the previously described MALI bioreactor system (Cei et al., 2020). The overall setup and geometry of the MALI system have not been changed. However, the following technical improvements were implemented. All of the components in contact with culture medium are now manufactured with PDMS-free material (namely polycarbonate, PC) to prevent potential artifacts due to leaching of toxicants from the PDMS into the cell culture medium. Moreover, an upgraded design of the PC holder more effectively prevents membrane slipping and leakage of culture medium during pressure oscillations required for inducing cyclic cell stretch as described below. The pressure sealing of the main chamber was improved and not only the pressure in the basal compartment (headspace of the medium reservoir, P_2_), but also the pressure in the apical compartment was measured continuously (P_1_) (Figure 1A) using two piezoresistive, monolithic silicon pressure transducers (MPX5050, Freescale Semiconductor, Munich, Germany).

A clinically used vibrating mesh nebulizer (Aeroneb Pro/Lab, Aerogen Inc., Galway, Ireland) is positioned at the top of the apical chamber for delivery of aerosolized substances to the cells on the BETA membrane. This type of nebulizer has liquid output rates and mass median droplet diameters ranging from 0.2 to 0.8 mL min^−1^ and 2.5 to 6 μm, respectively, depending on the specific type of nebulizer (Ding et al., 2020). Nebulization of 10 μL of liquid and subsequent spatially uniform deposition onto the cells cultured on the BETA membrane (5 cm^2^) with a deposition efficiency of 52% occurs within 2 min due to cloud settling (Cei et al., 2020). These aerosol delivery parameters are, independent of nebulizer performance in terms of droplet diameter or liquid output rate (Lenz et al., 2014) since cloud settling depends on the fractional aerosol volume in the air only and hence on the nebulized aerosol volume (10 μL) and the volume of the apical compartment of the chamber. Vibrating mesh nebulizers contain a porous membrane for aerosol production, which may be affected by cyclic positive pressure. Since aerosolized substance delivery is short (2 min) relative to typical cell-stretch experiments (>2 h), aerosolized substance application is typically decoupled from cyclic cell stretch, i.e., cell-stretch is not applied during aerosolization. This patented aerosol-cell exposure unit has recently been made commercially available as VITROCELL^®^Cloud MAX (VITROCELL Systems, Waldkirch, Germany), albeit only for standard transwell inserts, which cannot be subjected to cyclic stretch. When positive pressure is applied apically to the membrane (P_1_), the initially relaxed, flat, horizontally oriented membrane is stretched triaxially downwards expanding the volume of the apical compartment by a dome-shaped volume ΔV (Figures 1A, 4A). Due to the incompressible nature of water (culture medium), this change in apical volume reduces the air-filled headspace of the medium reservoir by ΔV and the corresponding increase in pressure (P_2_) can be directly related to the linear/area amplitude of membrane stretch (Figure 1A).

This special feature of the CIVIC bioreactor enables real-time monitoring of the experimental stretch parameters (amplitude, frequency) and Young's modulus of the cell-covered membrane with thickness t during triaxial stretch under wet conditions (contact with culture medium) by continuously monitoring the apical and basal pressures (P_1_ and P_2_, respectively). While the stretch frequency can be directly derived from the time course of P_1_ or P_2_, the linear (1D) and area (2D) amplitude as well as Young's modulus (elastic modulus; E, kPa) of the membrane can be determined from the maximal values of P_1_ and P_2_ according to the Equations (1)–(3) (Flory et al., 2007).

(1) $$\begin{array}{l} \Delta V=\Delta P\left(\frac{V_{0}}{P_{0}}\right),\;where\;\Delta P=P_{2}-P_{0} \end{array}$$

(2) $$\begin{array}{l} \Delta V=\pi \Delta h\;\left(\frac{a^{2}}{2}+\frac{\Delta h^{2}}{6}\right) \end{array}$$

(3) $$\begin{array}{l} \Delta P^{\prime }=\left(P_{1}-P_{2}\right)=\;\frac{4E\left(\frac{\Delta h}{a}\right)t}{3a\left({\left(\frac{\Delta h}{a}\right)}^{2}+1\right)}\left(1-\frac{1}{{\left(1+{\left(\frac{\Delta h}{a}\right)}^{2}\right)}^{3}}\right) \end{array}$$

Initially, the membrane is non-stretched and the pressure in both the apical and basal compartment is at ambient pressure P_0_ (on average 98.0 kPa in Munich, Germany) (see Figure 1A). Under these conditions, the radius (area) of the membrane a is 1.26 cm (5 cm^2^) and the headspace volume in the medium reservoir V_0_ is 30 mL (40 mL vessel filled with 10 mL medium). The deflection of the membrane perpendicular to the membrane (Δh) can be obtained from the corresponding change in apical/basal air volume ΔV, which is determined from Equations (1), (2) and the measured pressures P_1_ and P_2_. Young's modulus (elastic modulus; E, kPa) of the membrane can then be obtained from Equation (3), where t is the thickness of the membrane (ca. 5 μm; calculated by cross-sectional SEM analysis). For dome-shaped geometry (spherical cap), one can find the relative linear and area strain according to Equation (4).

(4) $$\begin{array}{l} \frac{\Delta L}{L}=\frac{\Delta h}{a}\;or\;\frac{\Delta S}{S}={\left(\frac{\Delta h}{a}\right)}^{2} \end{array}$$

The amplitude of cell stretch can be calculated from Equation (4), where Δh is the membrane deflection (0 ≤ Δh ≤ 0.11 cm) (Equations 1–3) and ΔS is membrane change in surface area during the stretch. P_1_ and P_2_ are 100.5 and 99.5 kPa for physiologic stretch (10% linear strain or 21% ΔS), respectively.

For optimum BETA membrane, the CIVIC bioreactor is able to apply a linear mechanical strain of up to 17% (or 3, which covers both physiologic and non-physiologic (overstretch) conditions. For those conditions, the optimum BETA membrane is resilient to 48 h cyclic stretch with no deformation, rupture, and creep.

### Membrane Fabrication

We recently introduced a novel ultra-thin co-polymeric membrane (BETA) transitioning from an initially stiff, hydrophilic, non-porous membrane to an elastic, porous substrate, providing optimum cell culture conditions during the two phases of typical *in vitro* alveolar cell-stretch experiments at the ALI (Doryab et al., 2020). Briefly, we employed a two-component (hybrid) polymeric material consisting of poly(ε-caprolactone) (PCL: Sigma-Aldrich, Mn 80,000) and gelatin (Type A from porcine skin, Sigma) chosen for their mechano-elastic and bioactivity properties, respectively. Different mass ratios of PCL and gelatin were dissolved in TFE [(2,2,2-trifluoroethanol) with >99.8% purity, Carl Roth GmbH, Karlsruhe, Germany] and stirred until the blend became homogenous. The PCL/gelatin mixture was then added to a custom-made spin-coater (2,000 rpm) to produce a thin film which was left to dry under vacuum (Figure 2A). The initially non-porous membrane (phase I: initial cell adhesion and growth) becomes gradually permeable (phase II: ALI culture) upon contact with the cell culture medium. The underlying concept of a biphasic membrane for cell-stretch experiments under ALI conditions mimicking the conditions in the alveolar tissue has been described in the introduction. The optimum concentrations of PCL and gelatin was determined previously as 9.35% PCL and 6.34% gelatin [w/v solvent]; solvent: ≥99% TFE (2,2,2-trifluoroethanol) (Doryab et al., 2020). Here, three new membranes with 15% PCL and 6, 8, and 10% of gelatin were manufactured. The membranes were placed in a holder for placement in the CIVIC system during cell-stretch experiments as described below (Figure 1C). Membranes were sterilized before cell culture experiments with ethanol and ultraviolet (UV) light exposure. The membrane is optically transparent and hence suitable for modern cell microscopy technologies.

### Membrane Characterization

The membranes were characterized in terms of thickness, ultrastructure, pore size, elemental and chemical composition, surface wettability, elastic modulus, 3D porosity, and cell proliferation (viability and cytotoxicity).

#### Physical, Elemental, and Chemical Characterization

Thickness, ultrastructure, and pore size of the membranes were analyzed by Scanning Electron Microscopy (SEM). The samples were fixed in 6% (v/v) glutaraldehyde (Sigma-Aldrich) and then dehydrated in gradient ethanol solutions followed by HDMS (hexamethyldisilazane, Sigma-Aldrich) for 15 min and subsequently mounted onto aluminum stubs, sputter-coated with platinum using Leica EM ACE600 vacuum coater, and imaged by SEM (Zeiss Crossbeam 340, Carl Zeiss AG, Oberkochen, Germany) with acceleration voltage of 2 kV. We also used Energy Dispersive X-ray Spectroscopy (EDS, X-max^N^, Oxford instruments) with an acceleration voltage of 8 kV to study qualitative elemental and the local distributions of certain elements (Carbon and Nitrogen) in the sample. Focused Ion Beam (FIB)/SEM (Zeiss Crossbeam 340, Carl Zeiss AG, Oberkochen, Germany) and FIB/SEM/EDS were employed to investigate the cross-sectional structures of the membranes at high resolution (30 kV; 700 pA and 1.5 nA). Surface roughness was assessed by an Atomic Force Microscope (AFM, Nanosurf Flex-Axiom) at room temperature. A scanning area of 80 μm was chosen. Scan rates of 0.5–0.15 Hz were used during mapping with 512 points per scan.

#### Surface Wettability (Water Contact Angle)

The surface wettability or Water Contact Angle (WCA) of the membranes was determined with the sessile drop method using an automated contact angle system OCA20 with an image processing system as described previously (Doryab et al., 2020).

#### Elastic Modulus (Young's Modulus)

Uniaxial (1D) tensile test (BOSE 5500 system, ElectroForce, Eden Prairie, MN, USA) with a load capacity of 22 N at a rate of 0.01 mm/s until rupture was used to calculate Young's modulus of the membrane (in phase I). Young's modulus of the membrane in wet (phase II) condition was measured using our novel pressure-based technique integrated into our CIVIC system described in more detail in Figure 1A.

#### Porosity

We used the liquid displacement method to measure the 3D porosity of the interconnected three-dimensional (3D) pore network of the membranes. Briefly, membranes were submerged in ethanol (EtOH, ≥99% purity) for 24 h. Gravimetric analysis prior to and after soaking the membrane with EtOH revealed the volume of EtOH (V_EToH_) inside the pores (V = m/ρ; *m*_*EtOH*_ = difference of mass prior to and after soaking; ρ_*EtOH*_ = 0.789 g cm^−3^) and the volume of the dry membrane (*V*_*m*_) [*m*_*m*_ = mass of membrane prior to soaking; ρ_*m*_ is the volume-weighted density of PCL (1.145 g cm^−3^) and gelatin (1.3 g cm^−3^)]. The empirical 3D porosity can then be calculated according to Equation (5).

(5) $$\begin{array}{l} Porosity=\;\frac{V_{EToH}}{V_{EToH}+V_{m}}. \end{array}$$

To account for EtOH adsorption on and/or microporosity of PCL itself, the apparent porosity of the pure PCL membrane (9.3 ± 1.7%; according to Equation 5) was subtracted from the measured porosity of the PCL/gelatin membranes.

Moreover, one can estimate the upper limit of porosity from the chemical composition of the membrane. Assuming gelatin has been completely dissolved in the culture medium (PCL is insoluble), one finds a theoretical upper limit for porosity from the volume fraction of gelatin based on Equation (6), where *V*_*g*_ and *V*_*PCL*_ are the volume fraction of gelatin and PCL in the composite of PCL/gelatin, respectively, and ρ_*g*_ and ρ_*PCL*_ are the density of gelatin and PCL (ρ_*g*_=1.30 g cm^−3^ and ρ_*PCL*_=1.45 g cm^−3^), respectively.

(6) $$\begin{array}{l} Theoretical\;Porosity=\;\frac{\;\frac{V_{g}}{\rho_{g}}}{\frac{V_{g}}{\rho_{g}}\;+\frac{\;V_{PCL}}{\rho_{PCL}}} \end{array}$$

#### Cell Proliferation, Morphology, and Cell Viability

Cell proliferation was assessed from the known number of cells seeded on the membrane (day 0) and the cells counted based on DAPI-stained (cell nucleus) CLSM images at the end of the submerged cell culture conditions (day 6). Moreover, cell proliferation was monitored indirectly with higher time-resolution by measuring cell viability in terms of a non-destructive metabolic activity assay (WST1, Roche, Mannheim, Germany), provided the metabolic activity of the cells is similar during the 6 days of cell growth. This test was performed on cell covered BETA membranes (1.3 cm^3^) and Corning^®^ Costar^®^ Transwell^®^ cell culture inserts (PET, 12-well, 3 μm pore), which was used as a commercial membrane to compare cell viability, cell number and morphology with that of the BETA membrane. Each membrane was incubated with 1 mL diluted WST1 reagent (1:15) at 37°C. After 15 min, 150 μL supernatant was transferred to a 96-well plate (4 times for each membrane) and absorbance was measured in a plate reader (Magellan™ Tecan) at 450 nm. All the results were normalized to the mean value of blank.

#### Lactate Dehydrogenase (LDH) Release

The cytotoxicity effect of the manufactured membranes was assessed by the detection of the release of lactate dehydrogenase (LDH; Roche Applied Science, Mannheim, Germany) from the cells, which indicates perforation of the cell membrane. According to the manufacturer's protocol, the determination of LDH activity was determined in the basal (and apical) medium by absorbance measurement at a wavelength of 492 nm. The LDH release is presented as the ratio of LDH dose (LDH concentration times medium volume) in the cell culture medium and the high control (cells treated with 2% [w/v] Triton X-100). Transwell inserts were used as a positive control since BETA membranes are more limited in supply than standard Transwell inserts. The LDH release for each membrane was normalized by the maximum possible LDH level (LDH contained in all cells).

### PDMS-Leached in Cell Culture Medium

We fabricated PDMS films for studying the leaching of PDMS oligomers into the culture medium. Briefly, the elastomers and crosslinker (1:10, Sylgard 184, Dow Corning) were mixed and degassed under vacuum. After casting, the film was cured in an oven at 60°C overnight. The PDMS film (thickness: 5 μm) was then cut using a standard biopsy punch (size: 5.0 mm; Kai medical, Solingen, Germany). The membranes were washed with PBS (three times) and disinfected using EtOH 80% and UV before immersing in the Dulbecco's Modified Eagle Medium: Nutrient Mixture F-12 (DMEM/F12, 1:1 v/v, Gibco) supplemented with 10% FCS (Gibco), 1% (v/v) Pen/Strep (100 U mL^−1^, Gibco), 1% L-glutamine (2 mM, Gibco). The PDMS punches and BETA membranes of the same size were soaked in 2 mL of culture medium for 2 days (the ratio of the surface area and the bulk volume of the membrane to the culture medium were 0.1 cm^2^ mL^−1^ and 4.9 × 10^−5^ cm^3^ mL^−1^).

The PDMS- and BETA-incubated media containing leached compounds were used to investigate their effect on cell viability with the WST1 assay. For this, A549 cells (1.5 × 10^5^ cells cm^−2^) were cultured under submerged conditions in 12-well multiwell plates using PDMS- and BETA membrane-leached media for up to 4 days and repeatedly analyzed for viability (WST1). As a control, A549 cells were seeded on the bottom of the well plate.

### Immunofluorescence

Cells were fixed in 4% paraformaldehyde (Sigma-Aldrich), washed with PBS and, permeabilized by 0.3% Triton X-100 (Sigma-Aldrich) in PBS at room temperature. To prevent any unspecific antibody binding, a blocking buffer (5% BSA and 0.1% TritonX-100) was added for 10 min. The cells were then incubated overnight at 4°C with Anti-E-Cadherin (mouse, 1:1,000; Invitrogen) and anti–ZO-1 monoclonal (mouse, 1:100; Invitrogen), in a blocking buffer (5% BSA and 0.1% TritonX-100). Cells were then incubated with secondary antibody Alexa Fluor^®^ 488 goat anti-mouse IgG (1:500; Invitrogen) and Alexa Fluor^®^ 555 goat anti-mouse IgG (1:500; Invitrogen). The F-actin cytoskeleton and cell nuclei were stained with Phalloidin 594 (1:40) and DAPI (1:100), respectively. The cells were then embedded in Glycergel (DAKO Schweiz AG, Baar, Switzerland). All cell images were acquired using a confocal laser scanning microscope (CLSM; LSM710, Carl Zeiss; Oberkochen, Germany) coupled to the Zen2009 software. For intensity quantification of particles, the images were recorded at five independent fields of view (region of interest: 134.95 × 134.95 μm) for each sample. The rectangular tool (Fiji) was used to measure the mean fluorescence intensity of background-subtracted images.

### TEER Measurement

Trans-epithelial electrical resistance (TEER) measurements of epithelial cells grown on the membrane were measured using the Millicell-ERS system (Millicell ERS-2, Millipore, USA). TEER is calculated by multiplying the cell-specific resistance (Ohm, Ω) and the effective surface area of the membrane (cm^2^). The TEER value of the blank BETA membrane was determined as 78 ± 10 (Ω cm^2^), which was then subtracted from the cell-covered membrane TEER values.

### Particokinetic Studies

For particle studies, we chose fluorescent amine-modified polystyrene (PS-NH_2_) spheres, fluorescent orange (Sigma-Aldrich, St. Louis, USA), with a mean diameter of 100 and 1,000 nm for particle study (Figure 4A) since amine-functionalized surfaces (positively charged particles) are associated with higher cellular uptake and internalization as compared to neutral or negatively charged ones (Rothen-Rutishauser et al., 2006; Zhu et al., 2013). A549 cells were grown on the optimized BETA membrane until confluence (submerged conditions for 6 days) and left for acclimatization at the ALI for 1 day. Particles were then nebulized directly onto the cells [deposited mass dose: 2.1 μg cm^−2^; surface area dose: 1.2 cm^2^ cm^−2^ (100 nm particles); 0.12 cm^2^ cm^−2^ (1,000 nm)] and incubate with the cells for 2 h under stretched or unstretched conditions.

For the unstretched experiments, the membranes were first put in special holders and placed in a 6-well plate which was then positioned in the aerosol-cell exposure chamber of a VITROCELL^®^Cloud 6 system (VITROCELL Systems, Waldkirch, Germany; aerosol exposed area: 146 cm^2^, deposition factor: 0.97), followed by nebulization of 250 μL of particle suspensions (1.25 mg mL^−1^ in 0.3% NaCl) with subsequent aerosol sedimentation onto the cells with 3 min as described by Lenz and colleagues (Lenz et al., 2014). Corning^®^ Costar^®^ Transwell^®^ cell culture inserts (6-well, PET membrane with 3 μm pores) were also used to compare transepithelial translocation of particles with cells cultured on the BETA membrane under unstretched and stretched conditions. For stretch experiments, particles are delivered to the cells using the nebulizer integrated in the CIVIC as described above (Figures 1A,B, 4A) with a known delivery efficiency of 52% (Doryab et al., 2020). Subsequently, a physiologic cyclic mechanical stretch (10% linear at 0.33 Hz) corresponding to respiratory conditions during light exercise was applied to the cells for 2 h.

The fractional particle transport across the epithelial barrier was determined by quantitative fluorescence spectroscopy of the culture media in the basal compartment of both unstretched and stretched treatment using a plate reader (Safire2™, Tecan; excitation: 520 nm, emission: 540 nm). For normalization to the cell-delivered dose a standard curve of the particle suspension in cell culture medium basal medium volume prepared and measured for fluorescence intensity. For the measurement of quantitative cellular uptake of particles with CLSM, the samples were washed with PBS to remove free or weakly adsorbed particles from the apical side of the cell layer. Subsequently, the cells on the membranes were fixed in 4% paraformaldehyde for CLSM analysis. The CLSM images (z-stacks) were then recorded at five randomly selected fields of view for each sample (*n* = 4; region of interest: 134.95 × 134.95 μm) and quantified for cumulative fluorescence intensity of z-stacks.

### Statistical Analysis

All data were analyzed using GraphPad Prism 8.4 (GraphPad Software, La Jolla, CA, USA). The details of each statistical analysis were presented in the caption of the figures.

## Data Availability Statement

The original contributions presented in the study are included in the article/Supplementary Material, further inquiries can be directed to the corresponding author/s.

## Author Contributions

AD and OS designed the experiments, analyzed the data, and wrote the manuscript. AD manufactured and characterized the BETA membrane and performed all the cell experiments and particle and particokinetic studies. AD, OS, and AS modified the CIVIC bioreactor systems. AD and PS performed SEM, FIB-SEM, and EDS-SEM. SO assisted AD with particle study of the Transwell^®^ insert under static conditions. JG, MT, TS, AH, CV, AA, and MR provided the input to data interpretation in their respective field of expertise and contributed to the writing of the manuscript. All authors contributed to the article and approved the submitted version.

## Conflict of Interest

OS declares that the aerosol-cell delivery methods described here are patent-protected and made commercially available by VITROCELL Systems under a license agreement with the Helmholtz Zentrum München. The remaining authors declare that the research was conducted in the absence of any commercial or financial relationships that could be construed as a potential conflict of interest.

## Supplementary Material

Supplementary Figure 1 The Water Contact Angle (WCA) of the PDMS and BETA membranes using an automated contact angle system OCA20 with an image processing system (mean ± SD).

Supplementary Figure 2 SEM micrograph of bronchial epithelial 16HBE14o^−^ cells grown on the membrane (cultured 6 days submerged and 24 h ALI culture). (Left) Confluent cell layer scale bar of overview and the magnified insert is 25 and 2 μm, respectively. (Right) Pseudocolored cells (using the GNU Image Manipulation Program (GIMP 2.10.8) (http://www.gimp.org/), showing cracks between cells, which were induced due to dehydration of the samples for SEM. The scale bar is 10 μm.

Supplementary Figure 3 No significant effect of 2 h physiologic stretch and particle exposure (diameter: 100 and 1,000 nm) on cell viability (WST1 assay; A549 cells) was observed. The viability data were normalized by the corresponding value of the Transwell inserts (no stretch) (Data are reported as mean ± SD. *n* = 3; Two-way ANOVA with Sidak test).

Supplementary Video 1 BETA membrane motion in the CIVIC bioreactor system when a cyclic mechanical stretch (linear strain: 10%; breathing/stretch frequency: 0.33 Hz) is applied to the membrane.
