# Supplementary figures and images for: A Bioinspired in vitro Lung Model to Study Particokinetics of Nano-/Microparticles Under Cyclic Stretch and Air-Liquid Interface Conditions
Source: Front Bioeng Biotechnol. 2021 Jan 29;9:616830. doi: 10.3389/fbioe.2021.616830 (PMC7902031; doi:10.3389/fbioe.2021.616830)

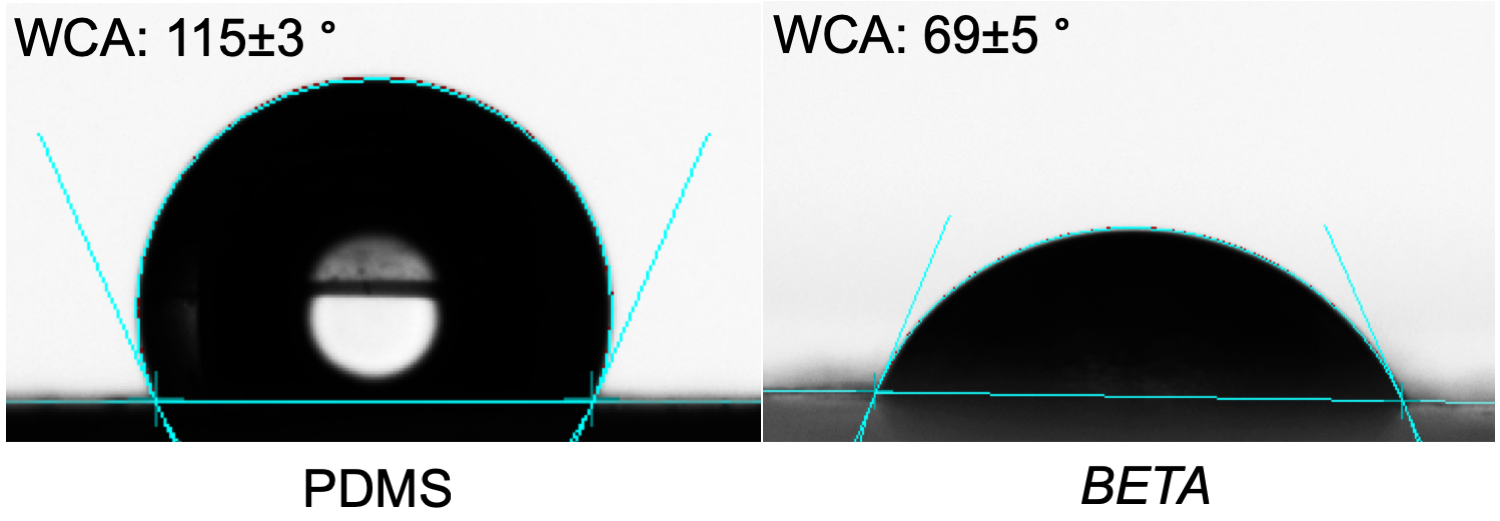

Supplement: Supplementary Figure 1 — The Water Contact Angle (WCA) of the PDMS and BETA membranes using an automated contact angle system OCA20 with an image processing system (mean ± SD). [file Image_1.TIFF]

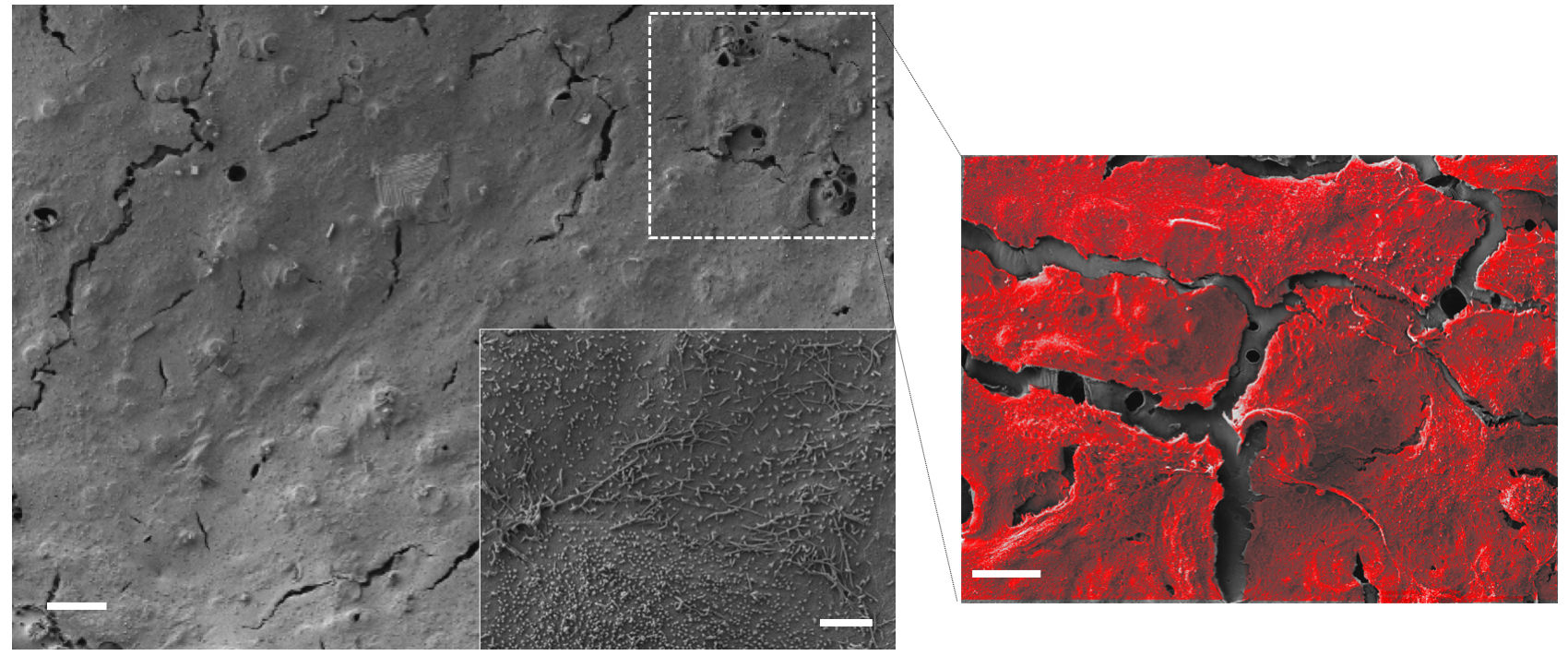

Supplement: Supplementary Figure 2 — SEM micrograph of bronchial epithelial 16HBE14o− cells grown on the membrane (cultured 6 days submerged and 24 h ALI culture). (Left) Confluent cell layer scale bar of overview and the magnified insert is 25 and 2 μm, respectively. (Right) Pseudocolored cells (using the GNU Image Manipulation Program (GIMP 2.10.8) (http://www.gimp.org/), showing cracks between cells, which were induced due to dehydration of the samples for SEM. The scale bar is 10 μm. [file Image_2.TIF]

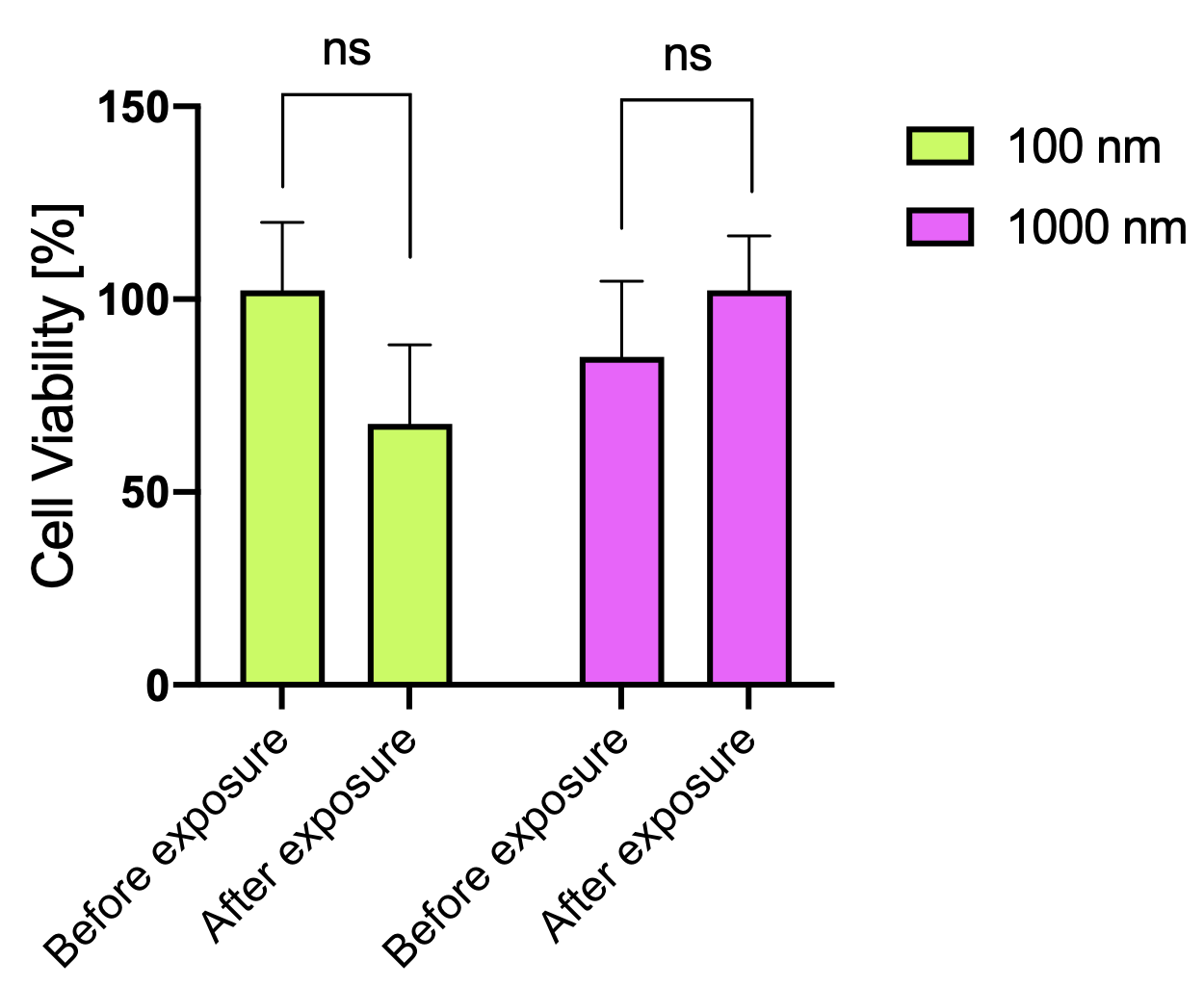

Supplement: Supplementary Figure 3 — No significant effect of 2 h physiologic stretch and particle exposure (diameter: 100 and 1,000 nm) on cell viability (WST1 assay; A549 cells) was observed. The viability data were normalized by the corresponding value of the Transwell inserts (no stretch) (Data are reported as mean ± SD. n = 3; Two-way ANOVA with Sidak test). [file Image_3.TIFF]
